# A mouse brain atlas based on dendritic microenvironments

**DOI:** 10.1038/s41593-025-02119-6

**Published:** 2025-11-24

**Authors:** Yufeng Liu, Sujun Zhao, Zhixi Yun, Feng Xiong, Hanchuan Peng

**Affiliations:** 1 New Cornerstone Science Laboratory, Institute for Brain and Intelligence, Fudan University, Shanghai, China; 2 New Cornerstone Science Laboratory, Institute for Brain and Intelligence, Southeast University, Nanjing, China; 3 Shanghai Academy of Natural Sciences (SANS), Fudan University, Shanghai, China

**Keywords:** Neuroscience, Computational biology and bioinformatics, Systems biology

## Abstract

Brain atlases map the spatial organization of neural tissue and serve as anatomical references. Current mouse brain atlases define regions based primarily on cell density patterns but overlook how neurons extend their branches (dendrites) to form local networks. Here we show that mapping dendrites enhanced by their local neighborhoods—which we call microenvironments—reveals a finer-grained brain organization. We analyzed dendrite patterns from more than 100,000 neurons across 111 mouse brains and discovered that neurons group into distinct microenvironments that subdivide known brain regions, nearly doubling the number of identifiable areas compared with the standard Allen Common Coordinate Framework. Remarkably, hippocampal neurons with similar local dendrite arrangements tend to form long-range connections to similar distant targets, suggesting that local structure predicts global connectivity. This microenvironment atlas complements existing resources by revealing previously hidden subdivisions and correlations that align with functional differences, offering new insights into how brain structure relates to function.

## Abstract (web summary)

The authors developed a mouse brain atlas using local dendritic morphological patterns from 101,136 neurons. This representation not only improves anatomical precision but also uncovers associations between local dendritic architecture and long-range neuronal projections.

## Main

Digital brain atlases are critical resources by providing a consistent anatomical framework for cross-brain mapping that facilitates the integration of diverse datasets and enables researchers to compare findings across studies. For example, the Allen Brain Atlas^1,2^ has been pivotal in advancing the understanding of gene expression patterns and their relationships to brain anatomy and function. The Common Coordinate Framework version 3 (CCFv3)^3^ serves as a valuable reference for aligning neuroimaging and neuroanatomical data. These atlases also spur the development of new hypotheses and the discovery of previously unobserved cell types and subtypes^4,5^.

Most existing mouse brain atlases are constructed upon collections of images capturing neuronal features such as histological stains, gene expression and connectivity, as seen in the Allen Reference Atlas^1^ and the Franklin-Paxinos atlas^6^, or combinations of multimodal features in CCFv3 (ref. ^3^). One of the major limitations of these approaches is the inadequate resolution in certain brain regions, making it difficult to capture fine-grained anatomical details. This challenge arises from the limited discriminative power of the utilized neuroanatomical features, such as cell density. Indeed, there has been debate among neuroanatomists on the accuracy and granularity of several CCF regions^7^, which call for independent validation using alternative labeling and annotation standards. This problem is even more obvious when various levels of anatomical deformation are introduced in different sample preparation methods used in developing these atlases, such as serial thick sections^8^, serial thin sections^9,10^ and other tissue-clearing techniques^11^ and tissue expansion methods^12^.

A valuable approach for constructing a high-resolution brain atlas is to incorporate more functionally informative features, such as single-cell transcriptomics. Single-cell spatial transcriptomic techniques such as 10x Genomics and multiplexed error-robust fluorescence in situ hybridization (MERFISH)^13^ have recently gained popularity in creating systematic cell atlases^14,15^. However, studies on the mouse brain have shown that the cellular organization revealed by MERFISH does not always align with the anatomical layout of the CCFv3 atlas^14^. Therefore, building a high-resolution brain atlas remains a challenge.

Here we provide an orthogonal approach to the above efforts. We notice that single-neuron morphology has long been recognized as a critical feature for determining neuron types and brain anatomy^16,17^. Although obtaining large-scale full morphologies remains difficult, dendritic morphologies are more readily available. However, dendritic morphologies are often stereotyped across populations, sparking debates about their ability to differentiate neuron types^18^. In our previous research, we quantified the stereotypy and diversity of dendritic features across cortical regions in the human brain^19^. We extended this approach by introducing a dendritic microenvironment representation for each neuron. In the present study, a neuron’s ‘microenvironment’ refers to a spatially enhanced ensemble of local neighboring cells, exemplified here by the spatially weighted summation of dendritic morphological features of neurons within a defined radius. This framework may encompass alternative implementations and incorporate diverse cellular characteristics, such as cell type composition, inter-neuronal distances, soma density, cell body size or vascular geometries, but is not limited to the dendritic features analyzed here. Using this representation, we developed whole-brain dendritic microenvironments using the three-dimensional dendritic morphologies of more than 100,000 neurons in mouse brains. By integrating the morphological features of neighboring neurons, these microenvironments enhance neuroanatomical discrimination and show correlations with axonal projection patterns. This framework also facilitates the creation of a high-resolution mouse brain atlas, CCF-ME (‘microenvironment’), enabling the characterization of brain anatomy, projection specificity and transcriptional patterns with greater granularity.

## Results

### A high-resolution dendritic microenvironment atlas of the mouse brain

**Fig. 1:**
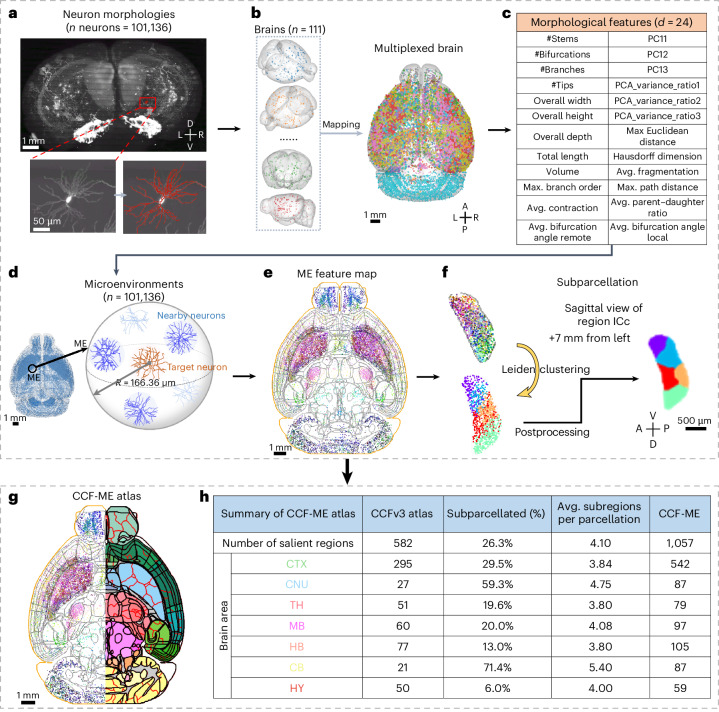
Overview of subparcellation and the CCF-ME atlas. **a**, Coronal view of a sparsely labeled mouse brain with an exemplar neuron highlighted. The autotraced morphology of the neuron is overlaid on the image (bottom right). L, left. **b**, A multiplexed brain containing all morphologies mapped from the original brains. Morphologies are colored according to the brains they come from. L, left. **c**, The morphological features used in this work (Methods). **d**, Construction of a microenvironment (ME). **e**, The horizontal middle slice with microenvironments within 0.5 mm projected onto the slice. Microenvironments are RGB colored by the top three morphological features identified using the minimal Redundancy Maximum Relevance algorithm. The region boundaries are outlined with gray dots; the outermost boundary of the brain is highlighted in orange. **f**, An example of subparcellation of the region ICc based on the microenvironment features. Different colors on the right mask indicate different subregions. **g**, Illustration of the CCF-ME atlas. Left: the left half of the horizontal middle slice of the microenvironment feature map. Right: CCF-ME, with new boundaries highlighted in red line. Brain regions are colored following CCFv3 convention. **h**, A summary table comparing CCF-ME and CCFv3. The column ‘Avg. subregions per parcellation’ shows the average number of subregions in CCF-ME identified for each subdivided CCF region. Only subparcellated CCF regions are considered when calculating ‘Avg. subregions per parcellation’. D, dorsal; V, ventral; R, right; A, anterior; P, posterior.

**Extended Data Fig. 1:**
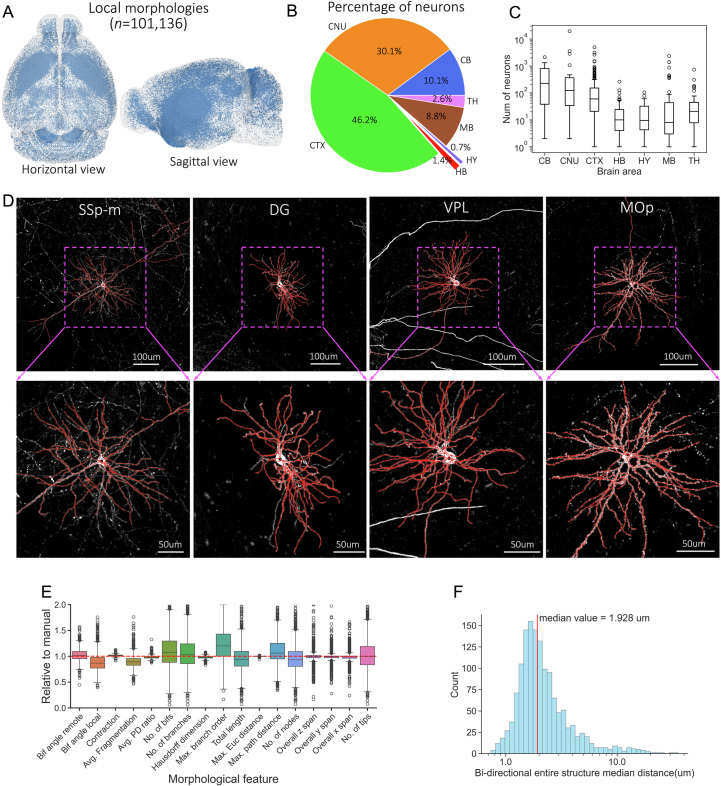
Summary of auto-traced morphologies. **A**. Horizontal and sagittal views of the 101,136 auto-reconstructed neurons mapped in the CCF atlas. **B**. Pie chart showing the distribution of neurons across the brain areas. **C**. Box plot of neuron number distribution within brain regions, based on 101,106 neurons from 7 areas. **D**. Maximum intensity projections (MIP) of four neuronal images with their corresponding auto-reconstructed morphologies overlaid. The skeletons of each morphology are colored in red. A zoomed-in view of each neuron is shown at the bottom. The soma regions for the neurons are annotated in white at the top. **E**. Box plot comparing morphological features of auto-reconstructed versus manually annotated neurons, based on 1,516 neurons present in both datasets. **F**. Histogram of the bi-directional median distance between auto-reconstructed and manually annotated morphologies, with a median value of 1.928 µm. Box plot: edges, 25th and 75th percentiles; central line, the median; whiskers, 1.5×the interquartile range of the edges; dots, outliers. Source data

We developed a high-resolution whole-brain atlas for the mouse by analyzing dendritic microenvironments, achieving greater granularity than existing brain atlases. This new atlas is based on the autoreconstruction of 101,136 neurons (Fig. 1a and Extended Data Fig. 1a) from 111 sparsely labeled fluorescence micro-optical sectioning tomography (fMOST) mouse brains^20^. Each neuron was reconstructed from an approximately 256-µm-width image volume centered on the cell body, capturing the majority of the dendrites. The intrinsic patterns embedded in dendritic subtrees, when enhanced by our proposed microenvironment representation, are sufficient to differentiate subregions within existing anatomical brain regions (as discussed later). These neurons span all seven major brain areas: cerebral cortex (CTX), cerebral nuclei (CNU), midbrain (MB), thalamus (TH), cerebellum (CB), hindbrain (HB) and hypothalamus (HY), with particularly high representation in the CTX (46.2%) and CNU (30.1%) (Extended Data Fig. 1b,c). These neurons span the majority of brain regions (528 out of 582 non-fiber tract, non-ventricle salient regions in the CCFv3 atlas), with an average of 192 neurons per region. Among them, 397 regions host at least 10 neurons each.

Each neuron was mapped to the CCFv3 atlas, placing them in a standardized isotropic coordinate space (Fig. 1b). For comparability, we extracted the soma-connected subtree within a 100-µm radius around the cell body for each neuron and represented it by a 24-dimensional morphological feature vector (Fig. 1c and Methods). The autotraced neurons (examples in Extended Data Fig. 1d) exhibit a median bidirectional distance of 1.928 µm compared to manual annotations (Extended Data Fig. 1f), with morphological features similar between the two methods, aside from minor variances (Extended Data Fig. 1e). Due to inherent differences in data generation and skeleton sparsity, local features may diverge between manual reconstructions and autotraced morphologies. These biases are evident in the slight decreases observed in local bifurcation angle (‘Avg. Bifurcation Angle Local’) and branch length (‘Avg. Fragmentation’) (Extended Data Fig. 1e). The small variances of these features indicate that they should be biased rather than erroneous (Extended Data Fig. 1e). We also provided 12 visual examples spanning six levels of reconstruction quality, defined based on total dendritic length and the number of branches (Supplementary Fig. 1). Even the worst cases (auto-to-manual ratio (*r*) = 0.7 or *r* = 1.2) closely resemble the manual annotations in overall shape and key local features (for example, branch length and straightness). Additionally, the pairwise distance pattern within autoreconstructions in feature space closely matches that of manually annotated data (Supplementary Fig. 2a), with a Spearman correlation coefficient of 0.8 (Supplementary Fig. 2b).

**Extended Data Fig. 2:**
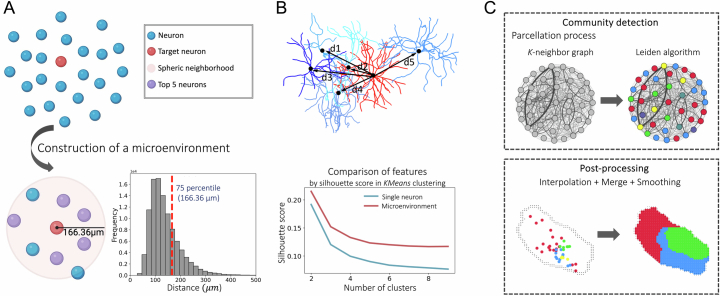
Subregion parcellation based on microenvironments. **A**. Diagram illustrating the construction of a microenvironment. A target neuron (red) is analyzed in the context of its local neighborhood (light pink; bottom). The target neuron identifies its six most similar neighbors (purple) within a 166.36 µm radius (see **Methods**). The histogram shows the frequency distribution of the distances between target neurons and their sixth nearest neurons. **B**. Schematic of the microenvironment feature calculation. A microenvironment is constructed by weighted summarization of the features of the six closest neurons (*i* = 0,1,2,3,4,5, where 0 means the target neuron itself). The line plot at the bottom displays the silhouette scores for feature separability of all neurons, as determined by K-Means clustering, across the number of clusters. **C**. Sub-parcellation of brain regions. At the initial community detection step, we construct a K-neighbor graph and use the Leiden algorithm to identify neuron communities. Then, all voxels in the target brain regions are estimated using nearest neighbor interpolation. The resulting subregions are iteratively merged and smoothed to get the final parcellation map. The final parcellation for the example region is illustrated at the bottom. Source data

**Extended Data Fig. 3:**
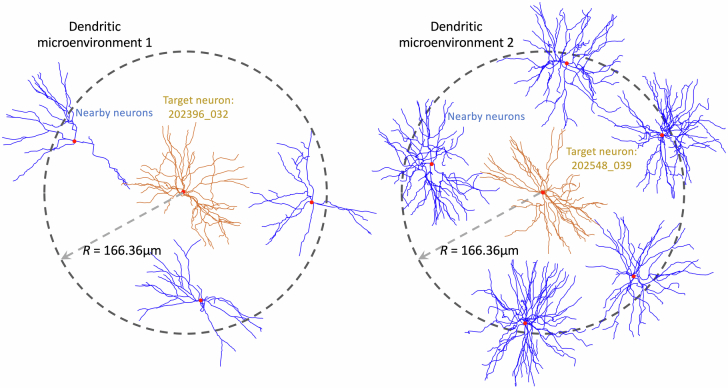
Illustration of two distinct microenvironments of similar target neurons. Target neurons are shown in chocolate, nearby neurons in blue, and somas in red.

We implemented a spatial-weighted summarization for microenvironment by integrating the morphological features of up to six neighboring neurons (Fig. 1d and Extended Data Fig. 2a). The implementation can vary, such as averaging neighboring neurons or direct concatenation of all neighboring neurons’ features. It can be further enhanced by incorporating diverse neuronal statistics, such as cell types or morphological variances (for example, Extended Data Fig. 3), thereby extending beyond dendritic morphology ensembling to optimize the capture of potential functional or developmental relationships among neurons. This representation is inspired by previous morpho-anatomical analyses of the human brain^19^. The microenvironments not only helped mitigate possible tracing flaws but also enhanced discriminative power between different neuron types, as demonstrated by higher silhouette scores for *k*-means clustering compared to single-neuron dendrites (Extended Data Fig. 2b) and higher cumulative variance ratios (Supplementary Fig. 3). Using the Leiden community detection algorithm, we classified microenvironments into communities based on their nearest neighbors, followed by postprocessing to generate smooth subregion boundaries (Extended Data Fig. 2c).

**Extended Data Fig. 4:**
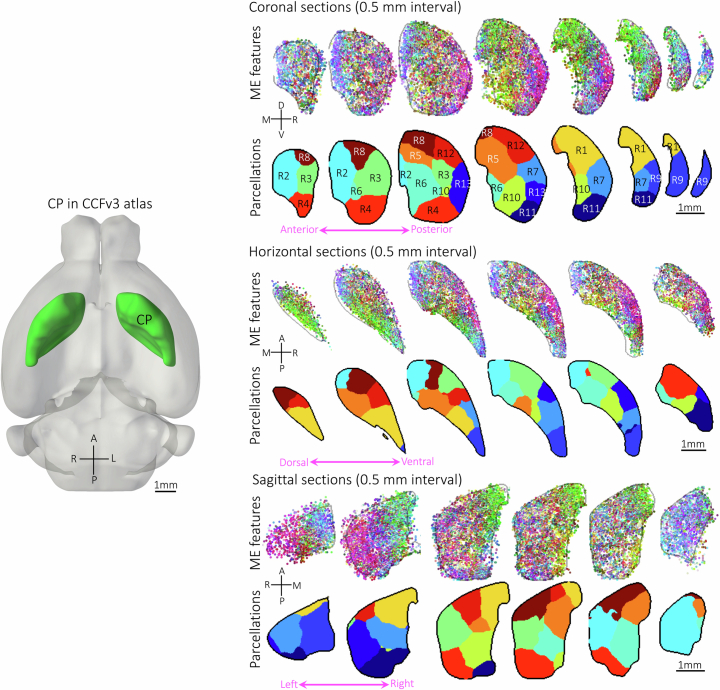
Microenvironment features and corresponding parcellations of the caudate putamen (CP). Coronal (top row), horizontal (middle row), and sagittal (bottom row) sections are sampled at 0.5 mm intervals. For visualization purposes, neurons within 25 µm of each section are mapped onto that section, thus microenvironments appearing outside the boundary are not actually out of bounds. A microenvironment is colored based on the values of the top three features, with the feature values assigned to the red, green, and blue channels of the corresponding voxel. Parcellated subregions are randomly colored.

**Extended Data Fig. 5:**
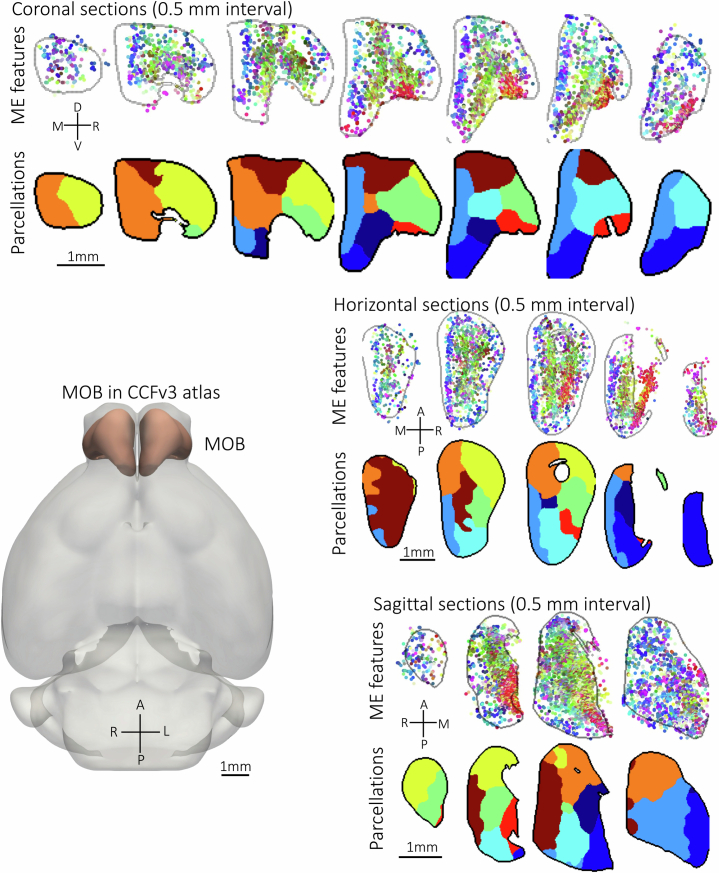
Microenvironment features and corresponding parcellations of the main olfactory bulb (MOB). Coronal (top row), horizontal (middle row), and sagittal (bottom row) sections are sampled at 0.5 mm intervals. For visualization purposes, neurons within 25 µm of each section are mapped onto that section, thus microenvironments appearing outside the boundary are not actually out of bounds. A microenvironment is colored based on the values of the top three features, with the feature values assigned to the red, green, and blue channels of the corresponding voxel. Parcellated subregions are randomly colored.

To explore morphological distributions across the brain, we created a whole-brain feature map using three key features: total neurite length (‘Total Length’), average straightness of branch (‘Avg. Contraction’) and branch length (‘Avg. Fragmentation’, defined as the number of 2-µm-long compartments in a branch). These features were selected using the minimal Redundancy Maximum Relevance^21^ algorithm. We color coded microenvironments based on these three features, assigning red, green and blue (RGB) values to each feature, respectively. The resulting map highlights spatial coherence and reveals inter-regional and intra-regional variations (Fig. 1e), such as the spatial differentiation observed in neurons of the inferior colliculus (ICc): ventral neurons exhibit longer total length (red points; Fig. 1f), and dorsal neurons show more straight branches (green points; Fig. 1f). As a result, ICc neurons are segmented into five subregions (Fig. 1f). More examples can be found in Extended Data Figs. 4 and 5.

**Extended Data Fig. 6:**
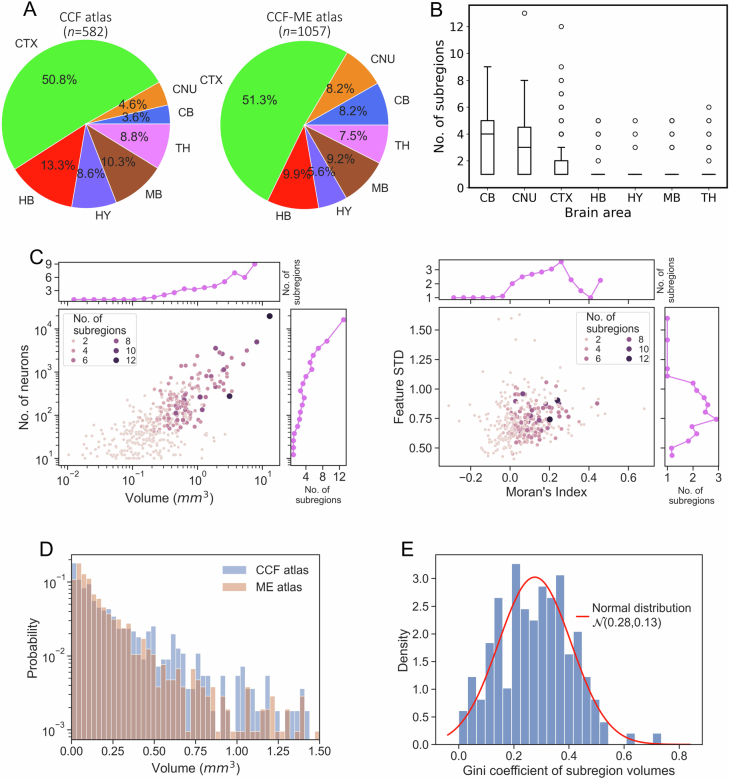
Summary of CCF-ME atlas. **A**. Pie charts showing the percentage of regions in different brain areas in the CCF atlas (*n* = 582) and microenvironment atlas (CCF-ME, *n* = 1057). **B**. Box plot showing the number of CCF-ME subregions per CCF region across different brain areas, using all 582 CCF regions except the “*root*” (*id* = 997). Box plot: edges, 25th and 75th percentiles; central line, the median; whiskers, 1.5×the interquartile range of the edges; dots, outliers. **C**. Relationship between the number of subregions and four metrics of each CCF region. Left, scatter plot of the region volume (“Volume”) and the number of neurons (“No. of neurons”) against the number of subregions. Both features are plotted in logarithmic space. Right, scatter plot of the standard deviation of microenvironment features (“Feature STD”) and spatial autocorrelation (“Moran’s Index”) against the number of subregions. **D**. Histograms of region volumes in the CCF atlas and ME atlas. The y-axis is presented on an exponential scale for enhanced visualization. **E**. Histogram showing the Gini coefficients of subregion volumes within parcellated CCF regions. A normal density function is fitted (μ = 0.28, σ = 0.13). Source data

From these dendritic microenvironments, we constructed a high-resolution atlas, CCF-ME, comprising 1,057 non-ventricular, non-fiber-tract salient regions—nearly double the 582 regions in CCFv3 (Fig. 1g). In CCF-ME, cortical regions remain consistent (51.3% versus 50.8%), whereas subregions in the CNU and CB increased substantially by 78% and 128%, respectively (Extended Data Fig. 6a), indicating a higher number of parcellated subregions in these brain areas (Extended Data Fig. 6b). Approximately 26.3% (*n* = 153) of CCF regions were subdivided into an average of 4.1 subregions (Fig. 1h). The regions in the CB exhibit the highest average number of subregions among common brain areas and a high ratio of region subparcellation (5.4 and 71.4%; Fig. 1g,h), whereas only 6% of HY regions are subparcellated (Fig. 1h). TH and HB regions show a slightly higher ratio of subparcellated CCF regions (19.6% and 13%; Fig. 1h).

We examined four metrics—number of neurons, volume, feature variability (‘Feature STD’) and spatial autocorrelation (‘Moran’s index’)—and found that volume and neuron count positively correlated with the number of subregions (Extended Data Fig. 6c, left). Nonlinear relationships were observed for feature variance and Moran’s index, where regions with intermediate values of these metrics exhibited the highest subregion counts (Extended Data Fig. 6c, right). This is reasonable as a small Moran’s index and feature variance value indicates a highly intermingled morphology distribution, and a large positive Moran’s index value represents a homogeneous distribution, both of which preclude the subdivision of a region. As a whole, the number of parcellated subregions correlates to all metrics considered but in different manners.

As expected, the regions in the CCF-ME atlas have smaller volumes (Extended Data Fig. 6d). The average volume in CCF-ME is 0.21 mm^3^, smaller than that in the CCF atlas (0.39 mm^3^). Only 3% of regions in CCF-ME have a volume larger than 1 mm^3^, compared to 8.6% in the CCFv3 atlas. The proportion of regions with a small volume (>0.027 mm^3^ but <0.2 mm^3^) increased from 40.9% in CCFv3 to 59.7% in CCF-ME (Extended Data Fig. 6d). For each CCF region that was divided into subregions, their volumes showed a modest uniformity, with a Gaussian distribution (*μ* = 0.28) of the Gini coefficients of subregion volumes (Extended Data Fig. 6e).

### Microenvironments improve local spatial coherence and reveal subregional differentiation

**Fig. 2:**
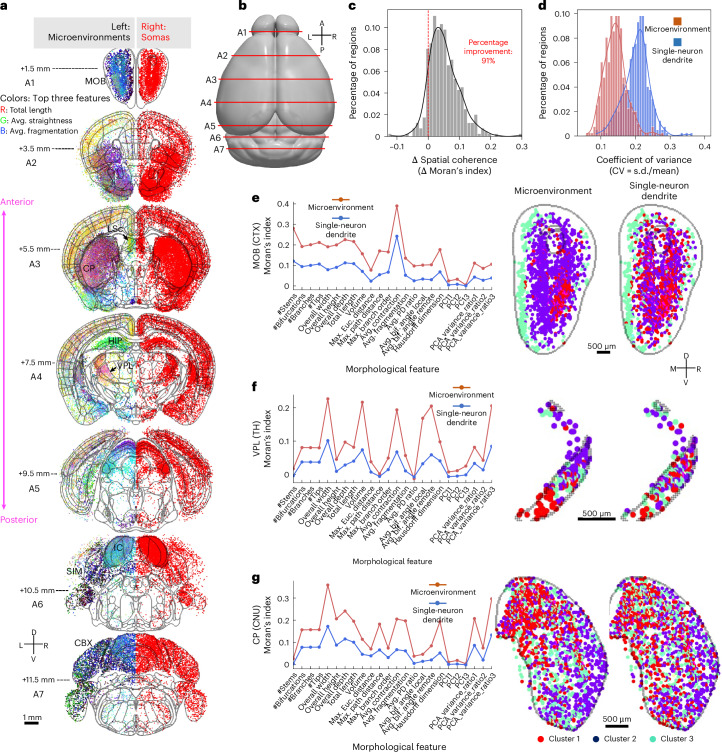
Microenvironment feature maps. **a**, Coronal slices of microenvironments (left) and somas (right) ranging from 1.5 mm to 11.5 mm along the anterior-posterior axis. A microenvironment is colored based on the values of the top three features, with the feature values assigned to the RGB channels of the corresponding voxel. To facilitate visualization, histogram equalization is applied to each channel. Somas and microenvironments within 0.5 mm of each slice are projected onto it using maximum intensity projection. The boundaries of each slice are highlighted by gray curves. Brain regions cited in the main text are annotated. L, left. Note that neurons within 0.5 mm on both sides are projected onto the median section, which may cause some neurons to fall outside the median section boundaries. These instances do not indicate registration errors. **b**, Illustration of the location of the seven slices on the CCFv3 atlas. L, left. **c**, The distribution of spatial coherence (calibrated by Moran’s index score) changes for CCF regions after using microenvironment representation. The vertical red dashed line highlights the location of non-improvement. **d**, The coefficients of variance distributions of single-neuron dendrites and microenvironments. **e**−**g**, Spatial coherence of morphological features (Moran’s index, first column), coronal slices of microenvironment feature maps (second column) and coronal slices of single-neuron dendrite feature maps (third column) for MOB (**e**), VPL (**f**) and CP (**g**). The feature maps are colored based on their clusters estimated using spectral clustering (*k* = 3) on the two-dimensional UMAP feature spaces. s.d., standard deviation. PD, parent–daughter. Source data

The large-scale dendritic reconstructions allow for detailed characterization of morphology and anatomical organization. By employing an ensemble-based microenvironment representation, the local coherence of morphological features was enhanced, revealing spatial differentiation throughout the brain. To illustrate, we compared the spatial distribution of microenvironments to soma distribution and single-neuron dendrite distribution (‘single-neuron dendrite’, which considers isolated dendritic features without incorporating their spatial context). We analyzed seven coronal slices ranging from 1.5 mm to 11.5 mm along the anterior-posterior axis (A1−A7; Fig. 2b). For each slice, neurons within 0.5 mm on either side of the anterior-posterior axis were color coded by the top three morphological features and projected onto the slice (Fig. 2a, left). We also mapped the soma distribution onto the same slices (Fig. 2a, right) for comparison. These cells were collected from 111 brains labeled by 30 reporter genes (Supplementary Table 1).

Microenvironments showed clear spatial preferences: neurons in the main olfactory bulb (MOB) had smaller total skeleton lengths and more curved branches (Fig. 2a1), whereas cortical neurons generally exhibited longer dendrites with straighter, shorter branches (Fig. 2a2−4). By contrast, hippocampal (HIP) neurons displayed straighter branches (Fig. 2a4), and distinct patterns emerged in regions such as the caudoputamen (CP), inferior colliculus (IC) and cerebellar cortex (CBX) (Fig. 2a3,a6,a7). For instance, IC neurons generally have straighter and longer branches, whereas those in surrounding regions, such as the simple lobular (SIM), have more curved branches (Supplementary Fig. 4c). Systematically, microenvironments generally showed similar dendritic microenvironments within the same brain area, such as the TH, where neurons had larger total local dendritic lengths and higher intra-area consistency (Supplementary Fig. 4a,b). Isocortical neurons display a dispersed total length distribution with modest branch length and straightness (Supplementary Fig. 4a), resulting in a smaller intra-area consistency (Supplementary Fig. 4b). Neurons of the CBX and CNU have more curved branches and smaller total lengths (Supplementary Fig. 4a), contributing to considerable intra-area and pairwise consistencies (Supplementary Fig. 4b). However, neurons within the same area can still display diverse morphologies, as seen in the CP and the caudal part of the lateral septal nucleus (LSc) from the striatum (Fig. 2a3).

Compared to single-neuron dendrites, microenvironments showed improved spatial coherence. Among 397 CCF regions with at least 10 neurons, 91% exhibited improved Moran’s index scores with microenvironments (Fig. 2c), along with reduced intra-region feature variances (Fig. 2d). This spatial coherence improvement was evident in clusters in regions such as the MOB in the cortex (Fig. 2e), the ventral posterolateral nucleus (VPL) in the TH (Fig. 2f) and the CP in the CNU (Fig. 2g), where microenvironments consistently showed higher feature coherence compared to individual neurons, uncovering spatial patterns that are otherwise obscured by single-neuron variability.

The microenvironments illustrated clear spatial differentiation within many CCF regions. In particular, microenvironments across various subregions of these regions displayed distinct morphological features, as evidenced by their spatial distributions in regions such as the MOB (Fig. 2a1 and Supplementary Fig. 4d), VPL (Fig. 2a4) and CP (Fig. 2a3). For clear visualization, we classified the microenvironments, and the resulting clusters exhibited clear spatial preferences across all regions (Fig. 2e−g). Another example is the nucleus accumbens (ACB), where neuronal morphologies were distributed in a structured manner (Supplementary Fig. 4d).

In summary, microenvironments enhance local spatial coherence of neuronal morphological layout while preserving spatial differentiation at the subregion level by integrating the morphological features of neighboring neurons. This approach allows the spatial organization of whole-brain morphologies to be inferred from microenvironments and facilitates subregional parcellations within established CCF brain regions.

### Microenvironments correlate with axonal projection of hippocampal neurons

**Extended Data Fig. 7:**
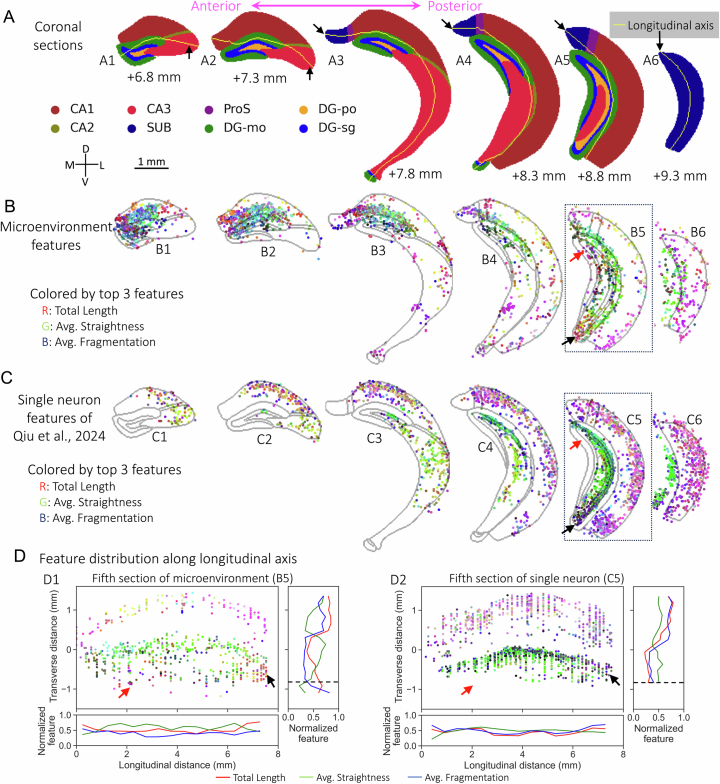
Spatial organization of the hippocampal neurons. **A**. Coronal views of masks for eight hippocampal regions. Six slices ranging from 6.8 to 9.3 mm (from the origin point in the CCF atlas) along the AP axis are shown. The longitudinal axis for each slice is highlighted by a yellow curve and pointed out by black arrows. The transverse axis is perpendicular to the longitudinal axis. D, V, M, and L: dorsal, ventral, medial, and lateral. **B-C**. The top three features of microenvironments (B) and manually annotated single neuron dendrites (C) are projected onto the slices. Neurons within 0.25 mm are mapped to the slice using maximum intensity projection. **D**. Feature distribution in the stretched space for the fifth slice of both microenvironments (left; D1) and single neuron dendrites (right; D2). The bottom and right insets of each component display the mean feature values of microenvironments along the longitudinal and transverse axes, respectively. The horizontal dashed black lines in the right insets indicate the lower limit along the transverse axis for all neurons in the single-neuron dataset. Source data

Given the spatial preference and subregional differentiation observed in microenvironments (Fig. 2), an important question arises: what is the relationship among dendritic morphologies, microenvironments and axonal projection patterns? Previous research reported that dendritic and axonal patterns can be consistent in some prefrontal cortical subtypes while divergent in others^22^. We aimed to explore this relationship using a recently released hippocampus dataset^23^, focusing on 3,822 neurons with dendrites reconstructed, and, at the same time, their somas were located within the eight HIP formation (HPF) regions: CA1, CA2, CA3, subiculum (SUB), prosubiculum (ProS) and the molecular, polymorph and granule cell layers of the dentate gyrus (DG-mo, DG-po and DG-sg) (Extended Data Fig. 7a).

**Fig. 3:**
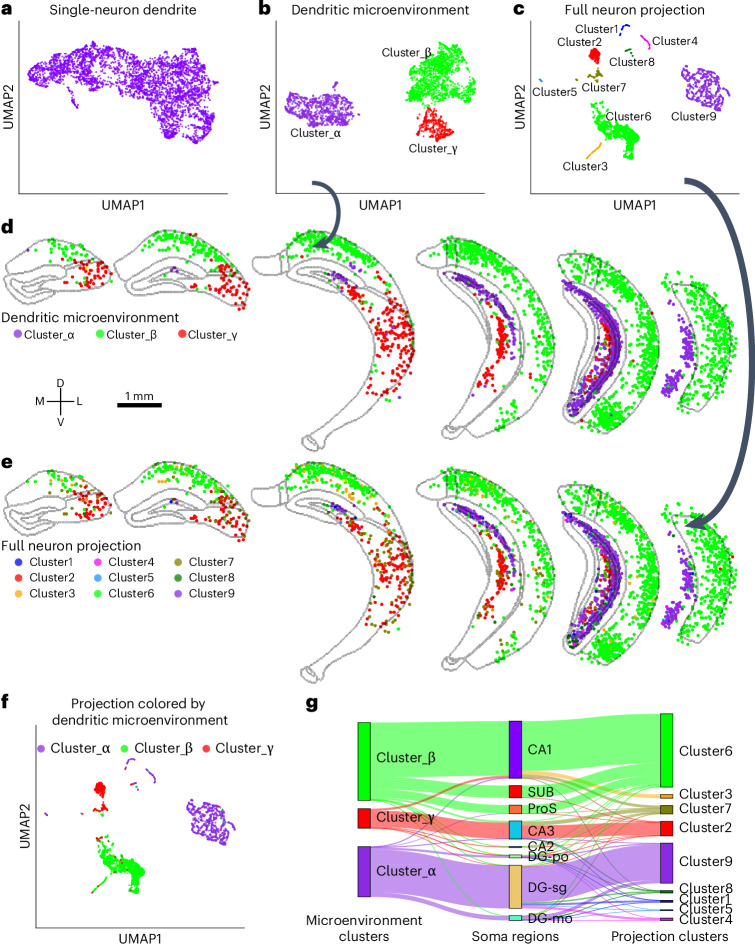
Correspondence between microenvironments and axonal projections of HPF neurons. **a**–**c**, UMAP visualization of clustering for single-neuron dendritic morphologies (**a**), dendritic microenvironments (**b**) and full neuron projections of HPF neurons (**c**), with projection intensity calculated as ln(length + 1), where length is the axonal shaft length per brain region (regions with length <1 mm excluded). Each dot represents a neuron, colored according to its cluster assignment. **d**,**e**, Coronal slices showing spatial distribution of microenvironment (**d**) and axonal projection (**e**) clusters, with region boundaries highlighted (region masks in Extended Data Fig. 7a). L, lateral; M, medial. **f**, UMAP of axonal projections colored by microenvironment cluster types. **g**, Sankey diagram linking microenvironment clusters, CCFv3 soma regions and axonal projection clusters.

The microenvironments of HPF neurons revealed three distinct clusters (α, β and γ; Fig. 3b), in contrast to the less distinguishable single-neuron dendritic morphologies (Fig. 3a), demonstrating a more divergent yet internally prototyped pattern when using the microenvironment representation. The spatial layout of these clusters aligns well with the CCFv3 HPF anatomy on all six coronal slices (Fig. 3d). Specifically, cluster γ neurons are primarily located in CA3; cluster α neurons are primarily located in DG-sg; and neurons in SUB, ProS and CA1 fall into cluster β (Fig. 3d,g).

Axonal projections are more divergent and categorized into nine clusters (clusters 1−9; Fig. 3c). The spatial distribution of projection clusters mirrored the dendritic microenvironments (Fig. 3d,e). Notably, projection clusters 6, 9 and 2 correspond to microenvironment clusters β, α and γ, respectively (Fig. 3f,g). Projection cluster 7 corresponds to both microenvironment clusters β and γ, without a strong bias toward either (Fig. 3f,g). This correspondence between dendritic microenvironments and axonal projections indicates that dendritic microenvironments may be capable of predicting long axonal projection patterns—a key insight given the difficulty of reconstructing long-projecting axons in large quantities.

**Extended Data Fig. 8:**
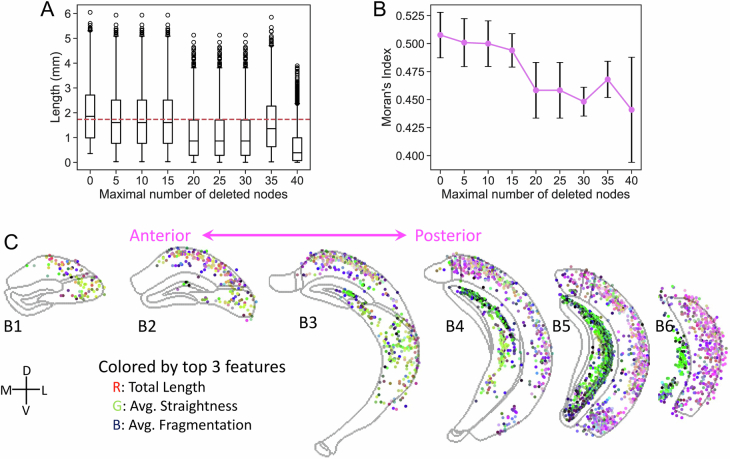
Robustness of spatial organization to morphological perturbations. **A**. Box plots illustrating the effect of randomly removing skeletal nodes on total skeleton length, with the dashed red line indicating the estimated median length of auto-traced local dendrites; 3,506 neurons with no NaN feature values across all perturbed datasets were used. Box plot: edges, 25th and 75th percentiles; central line, the median; whiskers, 1.5×the interquartile range of the edges; dots, outliers. **B**. Impact of node removal on spatial homogeneity, measured by Moran’s Index, with all downstream nodes connected to deleted nodes removed from the skeleton; the same 3,506 neurons as in **A** were analyzed. Data are presented as mean values ± SD. **C**. Coronal slices showing the spatial distribution of the top three morphological features across the anterior-posterior axis after perturbation by randomly removing up to 40 nodes and all downstream nodes. D, V, M, and L: dorsal, ventral, medial, and lateral. Source data

The finding should also hold for microenvironments constructed upon autotraced morphologies, which showed similar feature values and low reconstruction errors (Extended Data Fig. 1). At the same time, the spatial distribution of microenvironment features was consistent with that of manually annotated neurons in co-localized slices (slices B5 and B6; Extended Data Figs. 7b5,6 and 7c5,6). For instance, medial neurons were colored green, and lateral neurons appeared in magenta for both their microenvironments and the manually annotated neurons in slice 5 and slice 6 (Extended Data Figs. 7b5,6 and 7c5,6). In addition, the multidimensional spatial organization of HPF regions along the longitudinal and transverse axes is also reported in previous experiments utilizing genomic, anatomical and functional techniques^24–28^. Stretching the slices along the longitudinal axis further highlighted these layered differentiation and spatial patterns in both datasets (Extended Data Fig. 7d and Supplementary Fig. 5). Even when up to 40 skeletal compartments and subsequent branches (41% of the total skeleton length of a neuron) were removed, the spatial layout of microenvironments remained robust, with only minor decreases in Moran’s index from 0.51 to 0.44 (Extended Data Fig. 8). The deterioration is much more severe than in our autotraced local dendrites, which retained a median length of 93% compared to the manually annotated dendrites (dashed red line in Extended Data Figs. 1e and 8a). The resilience of spatial patterns, even under substantial perturbation, reinforces the robustness of the microenvironments based on autotraced morphologies.

### Microenvironments improve axonal projection mapping specificity

The correlation between microenvironments and axonal projection patterns in HPF neurons suggests that CCF-ME may enhance projection mapping specificity. To demonstrate, we compared the projection patterns of manually annotated HPF neurons^23^ across the CCFv3 atlas and our CCF-ME atlas.

**Fig. 4:**
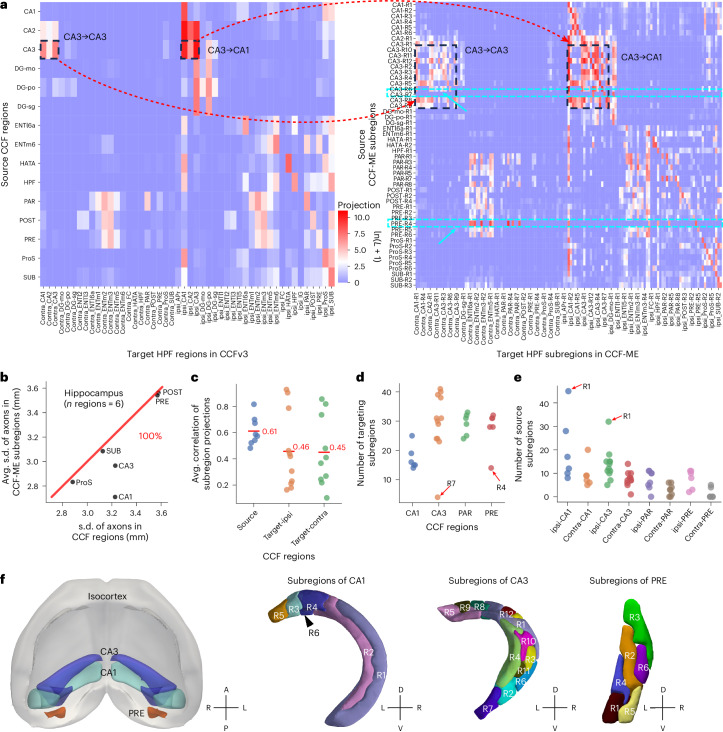
Axonal projection specificity of HPF neurons in the CCF-ME atlas. **a**, Projection matrix of HPF neurons across CCF-ME subregions in HPF, HY and striatum. The dashed black rectangles highlight the ipsilateral and contralateral projections of CA3 neurons to CA1 and CA3. The dashed cyan rectangles highlight the unique projection patterns for subregions CA3-R7 and PRE-R4. L, length. **b**, Comparison of the spatial standard deviations of axonal skeletons of all neurons in each CCF brain region with the average standard deviations of its corresponding CCF-ME subregions. **c**, Strip plot illustrating the distributions of average correlations between projections of subregions within each CCF region. ‘Source’, ‘Target-ipsi’ and ‘Target-contra’ represent the source CCF regions, ipsilateral target regions and contralateral target regions, respectively. The mean value for each type is indicated with a red line. **d**,**e**, Strip plots showing the distribution of the number of targeting subregions (**d**) and source subregions (**e**) for CA1, CA3, PAR and PRE subregions. Subregions within extreme numbers (R1 and R7 of CA3 and R1 and R4 of PRE) are explicitly annotated. **f**, Schematic illustration of regions CA1, CA3 and PRE in the context of isocortex in CCF (left) and the subparcellations of CA1 (second), CA3 (third) and PRE (right). Subregions are distinguished by different colors. L, left; HATA, hippocampo-amygdalar transition area; APr, area prostriata; POST, postsubiculum. Source data

Neurons from different CCF-ME subregions within the same CCF region exhibited distinct projection patterns. For example, neurons in ventral subregion R7 of CA3 showed lower overall projection intensity and projected exclusively to ipsilateral targets, including subregions CA1-R1, CA3-R2, CA3-R7 and DG-po-R1 (Fig. 4a, dashed cyan rectangles). By contrast, neurons from subregion R4 of the presubiculum (PRE) predominantly projected to contralateral HIP subregions (Fig. 4a, dashed cyan rectangles). Additionally, HPF neurons showed clear local projection preferences, characterized by enriched ipsilateral connections within HPF subregions (Fig. 4a). Other subregions, such as those in CA3, also exhibited unique, subregion-specific projection patterns (Fig. 4a). These findings highlight the increased specificity of projection mapping when using the CCF-ME atlas (Fig. 4a, right), which was not discernible in the CCFv3 atlas (Fig. 4a, left).

Quantitatively, neurons in each CCF region exhibited larger spatial variance than those in CCF-ME subregions, indicating that parcellating regions into CCF-ME subregions confers greater projection specificity (Fig. 4b). Correlation analyses further confirmed this specificity, with the average correlation between projections in source subregions and target subregions being 0.61 and approximately 0.45, respectively (Fig. 4c). The number of projected HPF subregions varied across subregions within the same CCF region, as demonstrated by the dispersed numbers in CA1, CA3, PAR and PRE subregions (Fig. 4d). A typical CA1 subregion targeted approximately 20 HPF subregions, whereas subregions in CA3, parasubiculum (PAR) and PRE were projected to roughly 30 subregions, with moderate diversity ranging from 20 to 45 (Fig. 4d). The R7 subregion of CA3 and the R4 subregion of PRE exhibited notably higher specificity (small numbers of targeted subregions; Fig. 4d). Variation was also evident in the number of input or source subregions for each CCF-ME subregion in hippocampus, especially between ipsilateral and contralateral hemispheres (Fig. 4e). Notably, CA1 and CA2 subregions tend to receive inputs from more subregions than other HPF regions, particularly ipsilaterally to CA1-R1 and CA3-R1 (Fig. 4e).

**Extended Data Fig. 9:**
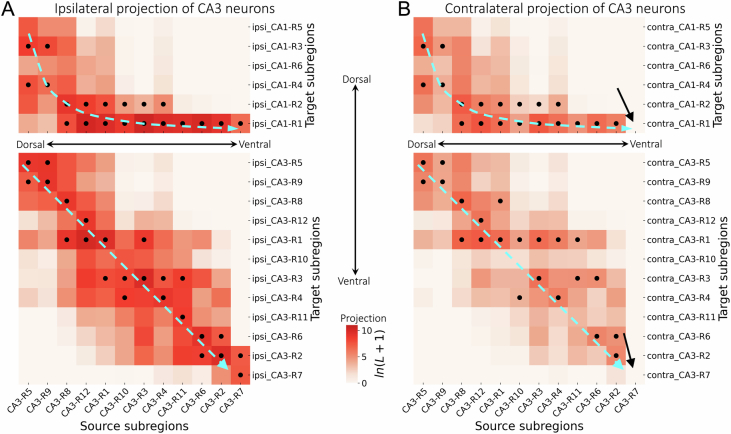
Ipsilateral (A) and contralateral (B) projection matrices of CA3 neurons. Subregions are sorted according to their locations from dorsal to ventral. Dots mark the top two subregions with the highest axonal skeleton lengths in these subregions. Dashed cyan lines highlight the trace of the top subregions from dorsal to ventral. Black arrows indicate the absent contralateral projections from the ventral subregion CA3-R7. Source data

The increased specificity in projection mapping enables an intuitive and detailed characterization of projection patterns such as the Schaffer collateral projections of CA3 neurons to CA1 (refs. ^23,29^) in both the ipsilateral and contralateral hemispheres, traversing the adjacent CA3 regions. Beyond the traditional collateral pattern reported previously, we also observed a mirrored but weaker projection into the contralateral hemisphere (Extended Data Fig. 9). Interestingly, neurons in the extreme ventral subregion R7 of CA3 project exclusively to the ventral subregions of both CA3 and CA1 in the ipsilateral hemisphere but with few contralateral projections (highlighted by black arrows in Extended Data Fig. 9).

In summary, the CCF-ME atlas supplements CCFv3 by providing specificity of projections of HPF neurons, such as the unique CA3 projection patterns of subregion R7.

### Dendritic microenvironments capture long-range connectivity specificity of CP neurons

In addition to linking dendritic microenvironments with the axonal projection patterns of HPF neurons, we investigated the connectivity specificity of the CCF-ME atlas and compared it with previous studies. First, we validated the subparcellation of CP by comparing it to previous parcellations derived from in-CP projections of cortical neurons^30,31^ and diverse projections of CP neurons^32^. Subsequently, we examined the subregion-specific input and output connectivity patterns of CP using a whole-brain single-neuron connectome.

Similar to the microenvironment features observed in the hippocampus (Extended Data Fig. 7b), CP neurons also exhibited multidimensional spatial differentiation (Extended Data Fig. 4). However, the boundaries between subpopulations were less clear than those in the hippocampus (Extended Data Fig. 7). Along the anterior-posterior axis, dorsal microenvironments displayed a gradual transition of morphologies to green colors (dorsal sides of the first six coronal slices; Extended Data Fig. 4). Subpopulations also emerged along the medial-lateral axis within coronal slices, such as the pink-colored subpopulations in the third, fourth and fifth coronal slices (Extended Data Fig. 4). Moreover, morphological differentiation along the dorsal-ventral axis was observed, as dorsal neurons in the fourth, fifth and sixth coronal slices exhibited varied features compared to ventral neurons (Extended Data Fig. 4).

**Fig. 5:**
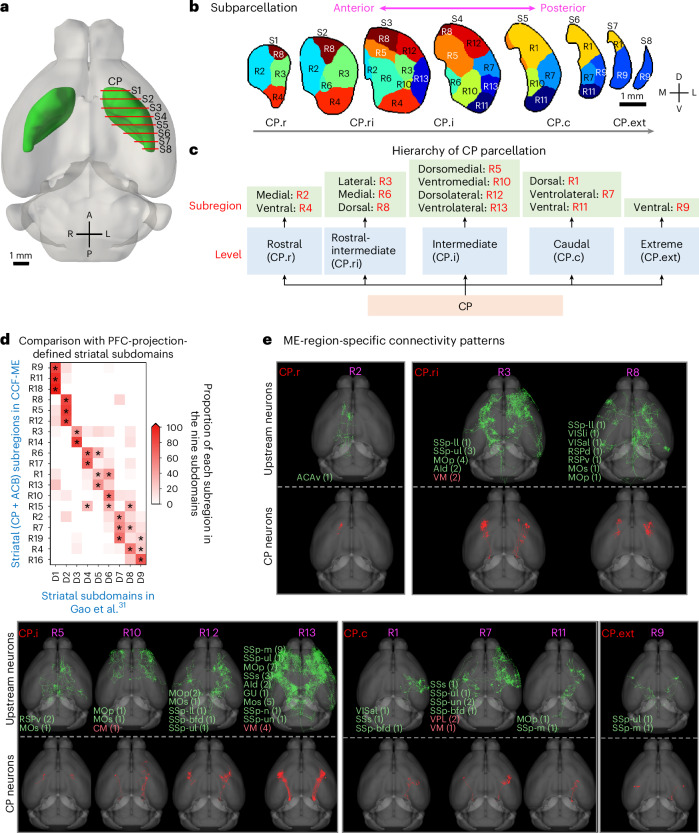
Subparcellation of CP and neuronal connectivity diversity. **a**, CP in CCFv3 atlas with eight evenly spaced slices (S1−S8) from anterior to posterior, marked by red lines (0.5 mm apart). L, left. **b**, CP subparcellation on eight slices, with subregions randomly colored. L, lateral. **c**, Anatomical hierarchy of CP subregions, highlighting putative locations (CP.r, CP.ri, CP.i, CP.c and CP.ext). **d**, Concordance of CCF-ME striatal subregions with prefrontal cortical neuron projection patterns, including CP and ACB for comparison; stars denote significant correspondences (*P* < 0.0001, one-sided Fisher’s exact test with Benjamini–Hochberg false discovery rate correction). **e**, Horizontal views of upstream and CP neurons for subregions, with upstream regions (color coded: green for CTX, tomato for TH) and neuron counts annotated in parentheses; only subregions with identified upstream neurons are shown (full abbreviations in Methods). PFC, prefrontal cortex. Source data

The differentiation of dendritic microenvironments and the resulting subparcellation align with the subdomain organization of the CP reported in studies on the cortico-striatal connectome and cortico-basal ganglia-thalamic networks^30,32^. Specifically, subregions in slice S1 are similar to those in the rostral level defined in ref. ^30^, whereas subregions in slices S3 and S4 align with those in the intermediate level, and subregions in the posterior slices S5 resemble those in the caudal level^30^. Previously, CP had been categorized into four levels along the anterior-posterior axis: rostral (CP.r), intermediate (CP.i), caudal (CP.c) and extreme (CP.ext)^30^. We extended this classification to five levels by adding a rostral intermediate (CP.ri) level to better describe the cross-slice three-dimensional subregions and mapped all 13 subregions into these five levels (Fig. 5c). Furthermore, the striatal subregions in CCF-ME are consistent with those identified based on the in-CP projection patterns of prefrontal cortical neurons^31^.

Connectivity analyses, using spatial distances between axonal boutons from 1,877 manually annotated full neuron morphologies and 18,370 dendritic morphologies across the brain, reveal subregion-specific patterns within the CP. Subregions receiving input from thalamic neurons are spatially clustered in the lateral areas of intermediate sections along the anterior-posterior axis (for example, R3, R7, R10 and R13 in Fig. 5b,e). The upstream neurons display morphological similarity in general (Fig. 5e), and CP neurons within these subregions also show similar morphology and projection patterns (Fig. 5e). By contrast, both CP projecting neurons and CP neurons from other subregions display subregion-specific preferences, with all analyzed neurons receiving input exclusively from cortical neurons, based on the available dataset.

### CCF-ME identifies complementary subregions of a MERFISH transcriptomic atlas

High-resolution transcriptomic techniques, especially recent spatial transcriptomics such as MERFISH^13^, have revolutionized neuronal cell typing at the whole-brain scale, providing potential fine-grained anatomical characterization. Using MERFISH, comprehensive cell atlases containing more than 300 subclasses and 5,000 clusters have been released^14,15^. However, the cell types identified are not always consistent with the existing CCF anatomy. Many region boundaries were missing in the transcriptomic modules, such as the boundary between layer 4 of the primary motor cortex and layer 4 of the primary somatosensory cortex^15^.

**Fig. 6:**
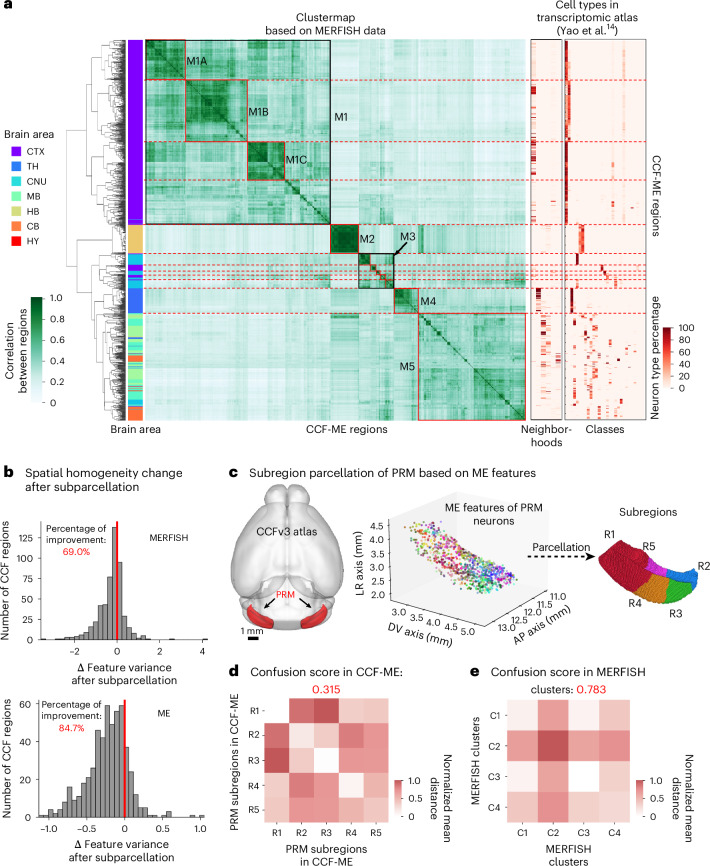
Comparison of microenvironments and MERFISH in identifying subregions. **a**, Clustermap of CCF-ME regions using MERFISH transcriptomics, with similarity defined as 1 minus normalized cosine distance of average log transcriptions; modules (M1−M5) and submodules (M1A−C and M3A−E) are outlined with solid squares and module boundaries in dashed red lines. **b**, Histogram of variance change after subparcellation in normalized feature space for MERFISH (top) and microenvironments (bottom), with percentage improvement in red and zero-change line marked. **c**, Subparcellation of PRM using microenvironment features, colored by top three features; subregions R1−R5 identified. **d**, Pairwise distance matrix of PRM subregions, showing normalized average distance between neuron pairs; confusion score at top. **e**, Pairwise distance matrix of four MERFISH PRM clusters, showing normalized Euclidean distance between neuron pairs; confusion score at top. AP, anterior-posterior; DV, dorsal-ventral; LR, left–right. Source data

We compared the CCF-ME with the state-of-the-art MERFISH transcriptomic data^14^. The clustering of MERFISH transcriptions across CCF-ME regions exhibited a modularized structure (Fig. 6a). Five modules and eight submodules were identified, encompassing all analyzed regions. Regions within most modules/submodules exhibited high homogeneity concerning anatomical brain areas as well as the neighborhoods and classes defined in previous work^14^. Specifically, the majority of cortical regions were clustered in module M1, distinguished by unique neighborhood and class types, except several olfactory regions, such as the MOB (Fig. 6a). Similarly, HB and TH regions displayed well-defined modular structures with distinct transcriptomic cell types (M2 and M4; Fig. 6a). Regions from other brain areas, including the CNU and HY, also exhibited stereotyped patterns (Fig. 6a). These findings indicate that the CCF-ME is overall consistent with the MERFISH data.

Interestingly, we found that 84.7% of CCF regions showed increased spatial homogeneity in the CCF-ME compared to that in the CCFv3 using the microenvironment data, in contrast to only 69% using MERFISH data (Fig. 6b and Methods). We also compared CCF-ME with the transcriptomic classes and observed that subregions within the same CCF region have a 28% probability of high transcriptomic concordance (*r* > 0.5; Supplementary Fig. 6). This finding confirms that CCF-ME not only captures regional variation consistent with known transcriptomic clustering but also provides finer-grained subregional delineations that can complement with other methods such as MERFISH.

**Extended Data Fig. 10:**
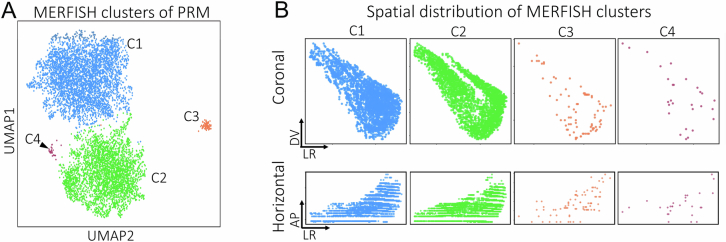
Intermingled spatial distribution of MERFISH clusters of PRM neurons. **A**. Scatter plot showing the four clusters for neurons in PRM based on MERFISH transcriptomic data. **B**. Coronal and horizontal views showing the spatial distribution of the four MERFISH clusters.

To investigate the biological indication of this finding, we further compared the spatial distributions of MERFISH data and microenvironments using the paramedian lobule (PRM) from the cerebellum as an example (Fig. 6c). The microenvironments exhibited clear differentiation along the longitudinal axis of the PRM (Fig. 6c). Neurons in each subregion showed smaller pairwise feature distances, except for neurons in subregion R5, which displayed moderate distances with both subregions R1 and R5 (Fig. 6d). By contrast, all four MERFISH clusters within the PRM were spatially intermingled and homogeneously distributed (Extended Data Fig. 10), as intra-cluster neuron pairs exhibited similar spatial distances to inter-cluster distances (Fig. 6e). Quantitatively, the confusion score among the five subregions of PRM in CCF-ME is 0.315, much smaller than the score between the four clusters of MERFISH data (0.783; Fig. 6e). In summary, the differentiated spatial preferences of microenvironments provide opportunities for higher-resolution anatomical identification.

Additionally, to demonstrate that these finer CCF-ME subregions capture distinct neuronal properties beyond what can be observed with spatial transcriptomics, we examined axonal projections of fully reconstructed neurons from three representative CCF regions: layer 2/3 of primary motor area (MOp2/3), mediodorsal nucleus of thalamus (MD) and piriform cortex (PIR). We present axonal projection patterns for each subregion (CCF-ME regions). Except the projection specificity among subregions, neurons from the same brain area tend to exhibit similar projection preferences across these subdivisions (Supplementary Fig. 7). For example, HPF neurons project predominantly to one specific subregion of MD (ID = 253) both ipsilaterally and contralaterally, whereas the ipsilateral projections to PIR subregions show moderate diversity. Thalamic neurons, by contrast, display notably different projection patterns to MOp2/3 relative to cortical neurons. These distinctions underscore the improved resolution provided by the microenvironment representation and further support the conclusion that CCF-ME can capture spatial neuronal properties at a finer scale.

## Discussion

The proposed CCF-ME atlas offers two main advantages over the current three-dimensional standard, CCFv3. First, it comprises 1,057 salient regions, an 80% increase, achieved by leveraging dendritic microenvironments from neighboring neurons to subparcellate CCFv3 regions. Second, because CCF-ME is built on top of the CCFv3 framework, it inherits established anatomical ontology and functional annotations.

The key technique involves microenvironment representation of dendritic arbors, which are more feasible to reconstruct than full single-neuron morphologies—a longstanding marker for cell typing^16^. Despite concerns about the ability of dendritic morphology to classify subtypes of cortical GABAergic interneurons^33^, our approach enhances local homogeneity and subregion differentiation within CCF regions (Fig. 2), as evidenced by improved clustering in the hippocampus compared to single-neuron dendrites (Fig. 3). This suggests that integrated neighbor information boosts discriminative power. However, our implementation is narrowly focused on dendritic structures, excluding other local features, such as cell types, inter-neuronal distances, soma density or vascular geometries, to isolate dendritic roles while limiting breadth. Future work could expand this by incorporating additional characteristics for brain organization insights.

Microenvironments also helped uncover hidden associations across different neuronal modalities. We found that the intrinsic microenvironment-related cell subtypes exhibit a strong correlation with their respective distal axonal projection patterns in the hippocampus, not seen in other previous analyses. The finding was further confirmed by higher projection mapping specificity and connectivity specificity when using the CCF-ME atlas (Figs. 4 and 5). These results suggest that studying dendritic microenvironments may offer valuable insights into functionally relevant axonal projection patterns, especially in large mammalian brains where full axonal reconstructions are not easily achievable. Whether the correlation is a universal principle across the whole brain or specific to certain areas, and to what extent these correlations exist in other areas, are intriguing follow-up questions to be clarified.

Spatial transcriptomic analyses have revolutionized systematic cell atlas research. However, analyses of these transcriptomic data revealed that many of these subclasses spatially overlap, precluding spatial subparcellation of the mouse brain (Fig. 6). By contrast, dendritic microenvironments exhibited either non-overlapping distributions or gradient differentiation for many CCF regions, providing a finer granularity of anatomical organization. This discrepancy may be attributed to the inadequate number of transcripts captured in current techniques or the inactivity of many region-specific genes at the current developmental stage. The spatial resolution achieved through dendritic microenvironment analysis highlights the potential for integrating morphological data with transcriptomic information to create a more comprehensive brain atlas. By combining these advanced methodologies, we can achieve a deeper understanding of brain organization and the functional implications of cellular diversity.

Morphologies in this study were generated via automated tracing, which may introduce minor inaccuracies in fine details but was validated against manual annotations for high-quality reconstructions (Extended Data Fig. 1 and Supplementary Figs. 2 and 3). The microenvironment representation also mitigated potential inaccuracies, and the spatial organization of microenvironments and parcellated subregions in the CP and hippocampus aligned with previous findings (Fig. 5 and Extended Data Figs. 4 and 7). To further mitigate potential inaccuracies inherent in automated reconstruction, we truncated the reconstructions to a 100-μm sphere around each cell body, yet they proved sufficiently discriminative to subdivide existing atlases into finer granularity. We acknowledge that the microenvironments of partial dendrites do not capture all the morphological features of complete dendritic arbors or full neurons. For instance, they may exclude highly discriminative apical and projection features, potentially restricting the granularity of parcellation. However, obtaining comprehensive reconstructions, particularly in human studies, remains challenging and limits the feasibility of large-scale whole-brain morphological analyses. Consequently, we focus on local dendrites, and our enhanced ‘microenvironment’ representation has proven effective for distinguishing subregional preferences in neuron distribution. As high-throughput whole-brain imaging and reconstruction techniques continue to advance, we anticipate the development of even more refined and accurate atlases.

Analyzing neurons across brains may be affected by brain-to-brain variability. Although they are less explored in healthy brains than condition-specific changes, they are likely less pronounced^34,35^. Aligning brains to the CCFv3 atlas using state-of-art registration tools produces registration errors, due to anatomical variations across individuals. For our microenvironment analysis, we mitigated this by (1) selecting the five most morphologically similar neurons instead of all neighboring ones, (2) applying spatial weighting to prioritize closer neurons and (3) subparcellating existing CCFv3 regions instead of the entire brain from scratch. These efforts minimize variability effect on the microenvironment construction and parcellation.

Morphological features such as dendritic contraction, volume, fragmentation and local angles are sensitive to variations in imaging resolution and reconstruction methods, potentially introducing biases in cross-dataset comparisons (see the local bifurcation angles and fragmentation in Extended Data Fig. 1e). Consistency improves among datasets with a shared pipeline, although variability persists due to varying imaging conditions. To address this, we standardized morphologies by mapping them to the standard CCFv3 space and resampling to a uniform fragmentation length. Although a uniform imaging pipeline offers the optimal solution, it is sometimes impractical in cross-laboratory collaborations. Resizing images to a consistent resolution could help, albeit with increased computational cost. Caution is advised when comparing local features across divergent tracing protocols.

## Methods

### Nomenclature and the CCFv3 atlas

Anatomical nomenclatures, abbreviations and ontology of the brain adhere to the CCFv3 standards. Throughout this paper, we used the 25-µm resolution version of CCFv3 (ref.^3^), which includes 671 brain regions. These regions consist of eight ventricle regions, 81 fiber tract regions and 582 salient regions. The salient regions, except one unclassified region (ID = 997), are distributed across seven major brain areas: CTX (*n* = 295), CNU (*n* = 27), CB (*n* = 21), HB (*n* = 77), HY (*n* = 50), MB (*n* = 60) and TH (*n* = 51). The full names of the region abbreviations are summarized in Supplementary Table 2.

### Local morphology tracing

The sparsely labeled fMOST mouse brains and the 182,497 somas are released in our previous work^20^. Most brains are accessible from the Brain Image Library (http://www.brainimagelibrary.org). The brains used in this work are listed in Supplementary Table 1, and detailed meta-information is documented in ref. ^20^.

The preprocessing and reconstruction protocols were similar to those in ref. ^20^, with the following updates. (1) We utilized 16-bit soma-centered highest-resolution image volumes of size 1,024 × 1,024 × 256 voxels (*x* × *y* × *z*; approximately 236 × 236 × 256 µm^3^) compared to the original 8-bit volumes in the second-highest resolution. (2) A robust image enhancement algorithm, NIEND^36^, was applied prior to neuron tracing. (3) Only all-path pruning (APP2)^37^ was used for neuron tracing, instead of both APP2 and neuTube^38^. (4) We optimized the postprocessing of the autotraced morphologies by adopting more conservative pruning criteria with a 45° angle and radius ratio of 2. In this way, we generated 179,568 reconstructions.

To obtain homogeneous skeletons, we registered the morphologies to the CCFv3 atlas using mBrainAligner^39,40^. The morphologies were spherically cropped around their soma locations with a radius of 100 µm, ensuring that each neuron was represented by its local reconstructions within an isotropic sphere. Skeletons that disconnected to their somas after cropping were removed. To standardize, we resampled the skeletons to ensure that the distance between two successive nodes on the same branch was 2 µm.

We filtered these reconstructions using two criteria: (1) the soma should be located in the 582 salient brain regions and (2) a good reconstruction must have similar morphological features to the feature distributions of manually annotated morphologies. To do so, morphologies that fell outside the 5% range of the minimum or maximum feature values of ‘Total Length’ and ‘#Bifurcations’ were discarded. The minimal and maximal feature values were calculated separately for different brain areas (for example, CTX). As such, we finally got 101,136 autotraced morphologies.

### Morphological feature extraction

Each neuron was represented by a 24-dimensional morphological feature vector. The first 18 features were calculated using the ‘global_neuron_feature’ plugin of Vaa3D^41,42^. These features are as follows: ‘#Stems’, ‘#Bifurcations’, ‘#Branches’, ‘#Tips’, ‘Overall Width’, ‘Overall Height’, ‘Overall Depth’, ‘Total Length’, ‘Volume’, ‘Max. Euclidean Distance’, ‘Max. Path Distance’, ‘Max. Branch Order’, ‘Avg. Contraction’, ‘Avg. Fragmentation’, ‘Avg. Parent-daughter Ratio’, ‘Avg. Bifurcation Angle Local’, ‘Avg. Bifurcation Angle Remote’ and ‘Hausdorff Dimension’. Six other features included the three values of the first principal component of the principal component analysis (PCA) of all skeletal nodes (PC11, PC12 and PC13) and the variance ratios of the three principal components. Each feature was *z*-score standardized separately when calculating distances or constructing a microenvironment.

### Morphology evaluation

Autotraced local dendritic morphologies were validated in two ways. First, we calculated a commonly used distance metric, bidirectional structure distance, between the traced morphologies and the dendrites of manually annotated morphologies within the same 100-µm spherical range. This metric estimates the average or median distance of all the nearest corresponding points (Extended Data Fig. 1f).

Second, we compared the morphological features of autotraced morphologies to those of annotated morphologies. To facilitate intuitive comparison, we calculated the relative feature values for all features, where a well-aligned reconstruction would show feature values close to 1. We then plotted a box-and-whisker plot with their statistics and outliers highlighted (Extended Data Fig. 1e).

Additionally, the quality of the reconstructions was evident in three ways. (1) Morphological features were relatively homogeneous within local neighborhoods but distinct across different regions and areas. (2) The microenvironment features of HPF neurons were consistent with those of manually annotated neurons in co-localized regions. (3) Subparcellations of striatum derived from the reconstructions aligned well with subdomain organizations reported in various studies.

### Microenvironment construction

A microenvironment is an ensembled representation of a target neuron and, at most, five spatially nearby neurons. For each target neuron, we first extracted all neurons within a local sphere (*R* = 166.36 µm) in the CCF space. The radius *R* was the 75th percentile of distances between the sixth nearest neuron and the target neuron for all neurons. The radius effectively balances robust spatial coherence with an optimal silhouette score for *k*-means clustering (Supplementary Fig. 8). If more than six neurons fell within radius *R*, the six neurons with the smallest feature space distances to the target neuron were selected. Otherwise, we kept all neurons within the distance *R*.

The construction of a microenvironment was done in the feature space. The feature vectors of neurons from the same microenvironment were integrated through spatial weighting. Specifically, each feature vector was weighted by the exponential of the negative relative distance to the target neuron (Extended Data Fig. 2a,b and equation (2)).

1 $$F=\mathop{\sum }\limits_{i=0}^{k}{w}_{i}\cdot {f}_{i};\,k\le 5$$

2 $${w}_{i}=\frac{\exp (-{d}_{i}/D)}{{\sum }_{i=0}^{k}\exp (-{d}_{i}/D)};\,D=166.36\,{\upmu}{\rm{m}}$$

where ${d}_{i}$ is the distance between the *i*th neuron and the target neuron; ${f}_{i}$ is the morphological feature vector; and *k* is the total number of neighboring neurons for the target neuron.

### Subregion parcellation

CCF brain regions were parcellated into subregions based on the spatial organization of microenvironment features. Regions with fewer than 40 neurons or smaller than 225 µm in any direction were skipped. For each eligible region, we constructed an undirected graph based on the spatial distances between microenvironments. A pair of ‘connected neurons’ is defined as two neurons where at least one is among the top *N* closest neighbors of the other based on spatial distance. The value of *N* was adaptively determined for each region using the silhouette score of the parcellation (see below). Given that many regions are layer differentiated, we rescaled the spatial distances according to the shape of each region when necessary, quantified using PCA of all voxels in the region. Edge weights were calculated as the exponential of the negative distance between the feature vectors of connected neurons. All nodes (microenvironments) in the graph were classified using the Leiden algorithm, followed by a majority voting community reassignment among the five nearest microenvironments.

The shape scaling started with performing PCA on the coordinates of the edge voxels of each brain region. After fitting the PCA model, we calculated the square roots of the eigenvalues (that is, the explained variances for the three principal components) and denoted them as an array *Α*. The scaling factor for each principal component was then computed as the sum of all square roots divided by each individual square root (that is, ${\rm{factors}}\left[i\right]=({\sum }_{0}^{2}{A}_{i})/{A}_{i}$). These scaling factors were then applied to the microenvironments in PCA-transformed space.

To obtain optimal clustering, we applied an adaptive parameterization strategy for community detection in each region based on silhouette scores. Two parameters were considered: the number of neurons used to initialize the graph and whether to apply shape-dependent scaling for laminar subregions. Results indicated that the best parameter combinations varied across regions. Specifically, shape-dependent spatial scaling was adopted for 73% of regions, whereas the number of neighbors showed greater variability, with 36% of regions utilizing the nearest neighbor count of 250 neurons. The clustering resulted in a 30% decrease in the average standard deviation of features compared to the original features.

The community for each voxel in the region was predicted using nearest-neighbor interpolation. The resulting parcellation was smoothed by two rounds of three-dimensional median filtering with a ball kernel of five voxels (125 µm) in radius. Subregions containing fewer than 512 voxels (0.008 mm^3^) were discarded and reassigned to nearby communities. During this process, we ensured that each connected component was treated as a subregion unless it was not a connected component in the original CCF atlas, thereby maintaining each subregion as an internally connected mask.

### Feature map visualization

We used the top three features of each neuron to intuitively visualize the spatial distribution of microenvironments. The top three features were identified with the minimal Redundancy Maximum Relevance algorithm, unless stated otherwise. The three features for the whole-brain feature map are the total length of neuronal skeletons (‘Total Length’), average straightness of branches (‘Avg. Contraction’ or ‘Avg. Straightness’) and average number of compartments within a branch (‘Avg. Fragmentation’, corresponds to branch length).

We mapped these features onto the CCF space according to their soma locations. For better visualization, each feature was first standardized using *z*-score normalization and then histogram equalized to enhance contrast and ensure a uniform distribution of intensities across the feature range of [0, 255], allowing for RGB color coding. Following the left−right convention, microenvironments in the right hemisphere were mirrored to the left hemisphere. This process produced a three-dimensional whole-brain feature map with 101,136 color-coded microenvironments. A similar protocol was applied for generating the feature maps of specific regions.

### HPF slice stretching

To obtain the longitudinal path for each HPF slice, we extracted the skeletons of the mask slices after performing a morphological closing using a square kernel of size five pixels (125 µm). The skeletons were pruned by iteratively removing the shortest branches until only one skeleton remained. This skeleton was then extended on both sides until it reached the boundaries of the mask. This method divided each HPF slice into three distinct components: background, dorsal (closer to the center of the bounding box) and ventral. The leftmost point of the path termini served as the origin of the new longitudinal-stretched coordinate space. To map all the microenvironments to the longitudinal-stretched space, we identified the nearest point on the longitudinal path as the anchor point for each microenvironment. The path distance from the anchor point to the origin point was used as the *x* coordinate, and the distance to the anchor point was used as the *y* coordinate. The dorsal part was assigned negative *y* coordinates, and the ventral part was assigned positive *y* coordinates.

The single-neuron reconstructions from HPF were utilized to compare the morphology distribution of manual reconstructions with our microenvironments. The manual reconstructions were released in ref. ^23^. A total of 3,822 neurons were retained for this comparison after excluding those without reconstructed dendrites and those located outside the eight regions of interest. For the evaluation of projection specificity among CCF-ME subregions, 10,023 neurons were used after removing neurons with more than one soma.

### Axonal projection mapping

To quantify the axonal projection, we calculated the projection as a vector with a dimensionality of *N*, where *N* corresponds to the number of targeted CCF brain regions or CCF-ME subregions. Each element of the projection vector corresponds to the logarithm of the total axonal length within a region or subregion. To avoid errors during the logarithmic transformation, 1 was added to the total length before applying the log function.

The projection calculation process began with the resampling of neuronal morphologies (stored in SWC format) into 2-µm-spaced skeletons using Vaa3D to ensure uniform sampling of the skeletons. Subsequently, the axonal components were extracted from the resampled SWC file. Given the uniform spacing, the total axonal length in each region or subregion can be estimated as the number of skeletal nodes multiplied by the spacing interval (2 µm). After calculating the axonal length in each region, we compiled these lengths into a projection vector, representing axonal projections across regions or subregions.

### Processing of MERFISH data

We utilized the MERFISH dataset (Zhuang-ABCA-1), publicly available at https://cellxgene.cziscience.com/collections/0cca8620-8dee-45d0-aef5-23f032a5cf09. It comprises 2.6 million spatial transcriptomic cells across 147 coronal sections, featuring a panel of 1,122 genes^15^. This dataset was classified into four nested levels—34 classes, 338 subclasses, 1,201 supertypes and 5,322 clusters—based on the transcriptomic and spatial atlas^14^.

Clustering of the raw MERFISH data followed the legacy workflow as follows. Preprocessing steps included removing low-quality cells and genes, normalizing total counts per cell, applying log transformation, selecting highly variable genes and scaling to unit variance. After PCA and the construction of a neighbor graph, we generated uniform manifold approximation and projections (UMAPs) and performed Leiden clustering with a resolution parameter set to 0.2.

To assess spatial coherence within brain regions, we used two metrics: feature variance and confusion score. Feature variance was calculated by averaging the standard deviations of the top three principal components within a specific brain region, indicating how varied the features were within a specific region. Regarding the confusion score, we first calculated a pairwise distance matrix, where each element represented the average similarity or distance between all intra-subregion or inter-subregion neuron pairs. Then, the confusion score was calculated as the ratio of off-diagonal to diagonal values in the confusion matrix. A lower confusion score reflects greater dissimilarity between subregions. For CCF-ME, the similarity is quantified as the average microenvironment feature distance between neurons. For MERFISH, each element in the matrix represents the average Euclidean distance among neurons within or between clusters (Fig. 6e).

### Identification of upstream neurons to CP neurons

The connectivity specificity of CP subregions was estimated based on a connectivity matrix defined in a single-neuron connectome study^43^. This matrix was constructed from 1,877 manually annotated single-neuron morphologies and 18,370 automatically reconstructed dendritic morphologies. Only neuron pairs with a connection score greater than 0.4 were identified as a true connection, to rule out possible false-positive connections.

### Statistics and reproducibility

No statistical methods were used to predetermine sample sizes. Neurons were reconstructed from multiple mouse brains, and 101,136 passing quality control were used for microenvironment reconstruction. For region parcellation, only regions with more than 40 neurons were included to ensure data stability. Regional projection analyses considered only brain regions or subregions with at least five neurons. Sample sizes for other calculations and analyses were maximized using available data. A random subset of 5,000 CP neurons (seed = 1,024) was sampled to evaluate Moran’s index in Fig. 2g for computational efficiency. Randomization was not applicable to other analyses.

### Reporting summary

Further information on research design is available in the Nature Portfolio Reporting Summary linked to this article.

## Online content

Any methods, additional references, Nature Portfolio reporting summaries, source data, extended data, supplementary information, acknowledgements, peer review information; details of author contributions and competing interests; and statements of data and code availability are available at 10.1038/s41593-025-02119-6.

## Supplementary information

Supplementary Information Supplementary Figs. 1−8

Reporting Summary

Supplementary Tables 1 and 2 The meta of brains (Supplementary Table 1, sheet 1) and region abbreviations (Supplementary Table 2, sheet 2).

## Source data

Source Data Fig. 2 Statistical source data.

Source Data Fig. 4 Statistical source data.

Source Data Fig. 5 Statistical source data.

Source Data Fig. 6 Statistical source data.

Source Data Extended Data Fig. 1 Statistical source data.

Source Data Extended Data Fig. 2 Statistical source data.

Source Data Extended Data Fig. 6 Statistical source data.

Source Data Extended Data Fig. 7 Statistical source data.

Source Data Extended Data Fig. 8 Statistical source data.

Source Data Extended Data Fig. 9 Statistical source data.

## Data Availability

The reconstructed morphologies and CCF-ME atlas are accessible via Zenodo at https://zenodo.org/records/13761460 (ref. ^44^). Source data are provided with this paper.

## Code availability

The source codes for neuron tracing, evaluation, microenvironment construction and analyses are available at https://github.com/SEU-ALLEN-codebase/BrainParcellation. The project was built upon our analytical library for neuron morphology, pylib (https://github.com/SEU-ALLEN-codebase/pylib). Other dependencies are summarized in the requirement.txt, which can be easily installed through the Python package installer (PIP). Morphological feature extraction was performed using the ‘global_neuron_feature’ plugin of Vaa3D (versions Vaa3D-x 1.1.4 and Vaa3D 4.001). Skeleton resampling was done using the ‘resample_swc’ plugin of Vaa3D (version Vaa3D-x 1.1.4). Short segment pruning was conducted using the ‘pruning_swc_simple’ plugin of Vaa3D (version Vaa3D-x 1.1.4). Feature selection was conducted using pymrmr (version 0.1.1). The clustering of microenvironments for each region was done using the Leiden algorithm (leidenalg; version 0.10.2). Connected component estimation was carried out with cc3d (version 3.12.4).
