# Supplementary material for: A mouse brain atlas based on dendritic microenvironments
Source: Nat Neurosci. 2025 Nov 24;29(1):111–22. doi: 10.1038/s41593-025-02119-6 (PMC12779555; doi:10.1038/s41593-025-02119-6)
Supplement: Supplementary file 1 — Supplementary Figs. 1−8 [file 41593_2025_2119_MOESM1_ESM.pdf]

nature neuroscience

Supplementary information https://doi.org/10.1038/s41593-025-02119-6

A mouse brain atlas based on dendritic microenvironments

In the format provided by the authors and unedited

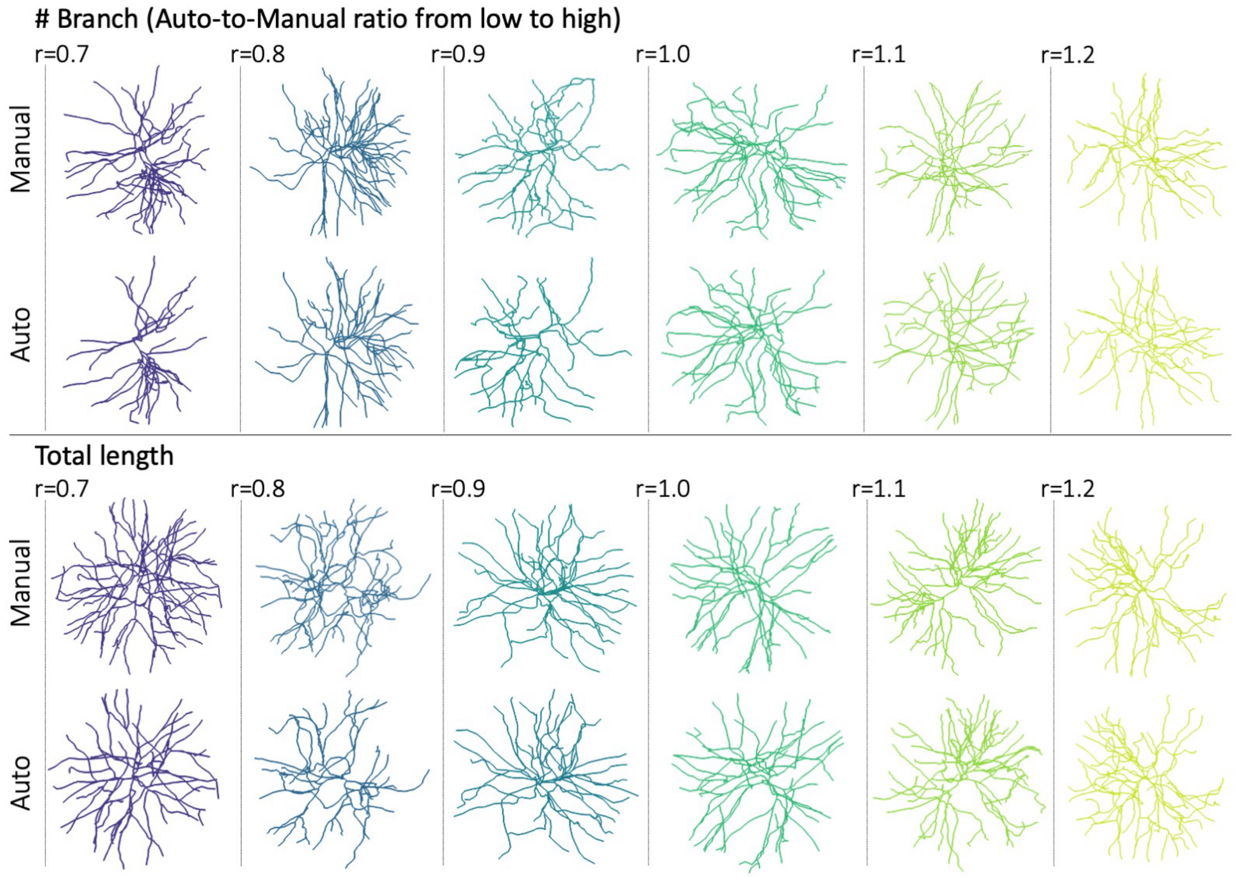

Supplementary Figure S1. Examples illustrating neuron reconstructions of varying quality. Each neuron's automated reconstruction and corresponding manual annotation are shown after cropping to a 100 μm sphere centered on the soma. The quality is evaluated by the ratio (*r*) of two key morphological features (the number of branches and total skeletal length) in the automated reconstruction compared with the manual annotation. The panels demonstrate how variations in r (from 0.7 to 1.2) affect both the automated and manual reconstructions.

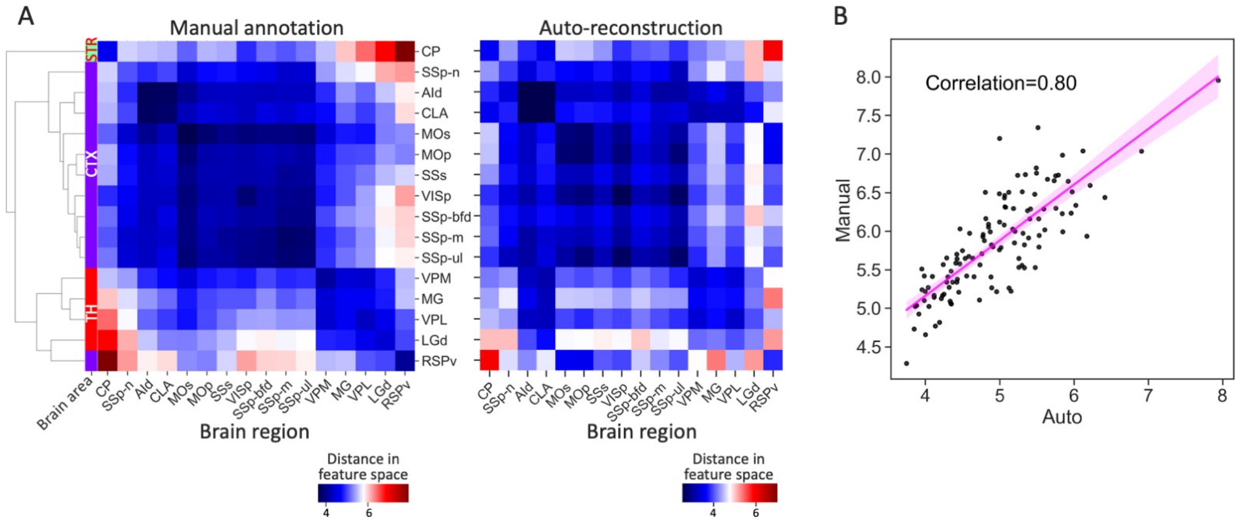

Supplementary Figure S2. **A**. Heatmaps comparing the morphological distance patterns among different brain regions in manual annotations (left) and auto-reconstructions (right). The color scale represents distance in feature space, and the left margin indicates the corresponding major brain areas (thalamus [TH], cortex [CTX], striatum [STR]). **B**. Scatter plot illustrating the correlation (Pearson's r = 0.80) between region-region feature distances derived from auto-reconstructions and those from manual annotations.

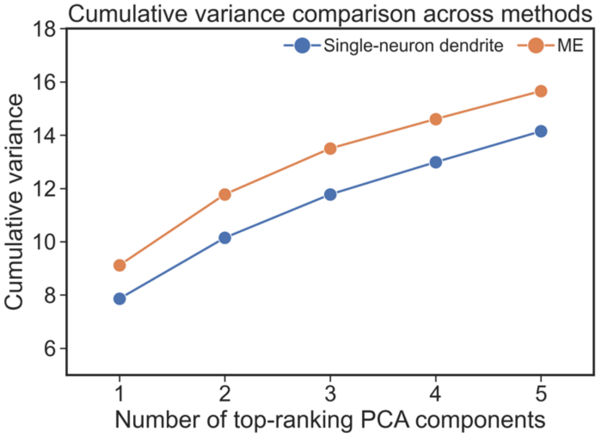

Supplementary Figure S3. Comparison of cumulative variance between single-neuron dendrite and microenvironment.

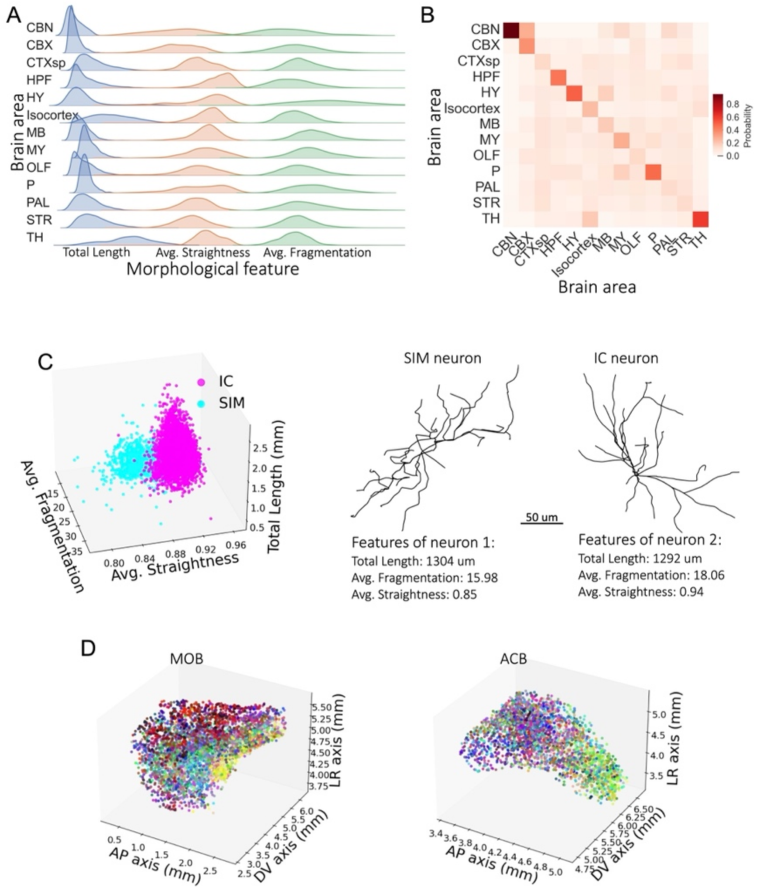

Supplementary Figure S4. Morphological differentiation across anatomical scales. A. Ridge plots showing the distributions of the top three features for 13 brain areas. The abbreviations for these structures follow the CCFv3 nomenclature: cerebellar nuclei (CBN), cerebellar cortex (CBX), cortical subplate (CTXsp), hippocampal formation (HPF), hypothalamus (HY), isocortex, midbrain (MB), medulla (MY), olfactory area (OLF), pons (P), pallidum (PAL), striatum (STR), and thalamus (TH). **B**. Heatmap of the pairwise feature similarity, calculated as the probability that the most similar microenvironment of each microenvironment belongs to a brain area. **C**. Left, the microenvironment feature distribution of the inferior colliculus (IC) neurons and simple lobular (SIM) neurons. Right, skeletal representations of two exemplar neurons from the IC and SIM, respectively, with their top three feature values annotated at the bottom. **D**. Feature landscapes of the main olfactory bulb (MOB) and nucleus accumbens (ACB) in the 3D feature space. The top three features were utilized for visualization. The features were histogram-equalized by channels and were standardized by each region.

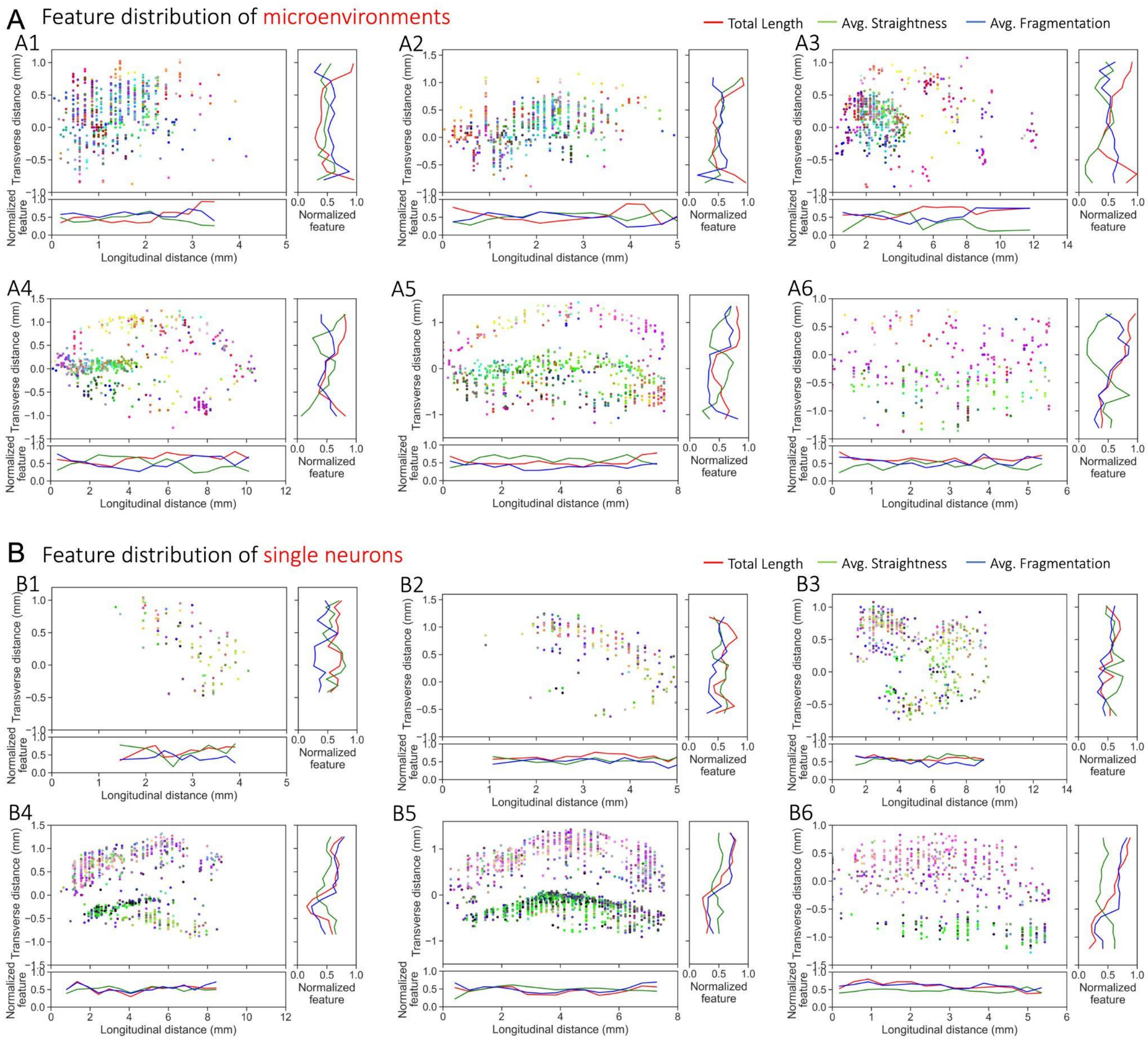

Supplementary Figure S5. Morphological feature distribution in the stretched space of hippocampal slices. The stretching of a slice is estimated by mapping each microenvironment to a new coordinate system according to the point with the minimal distance along the longitudinal axis (see **Methods**). The bottom and right insets of each component show the mean feature values of microenvironments along the longitudinal and transverse axes. **A-B**. Feature distribution for microenvironments (A) and manually annotated single neuron dendrites (B).

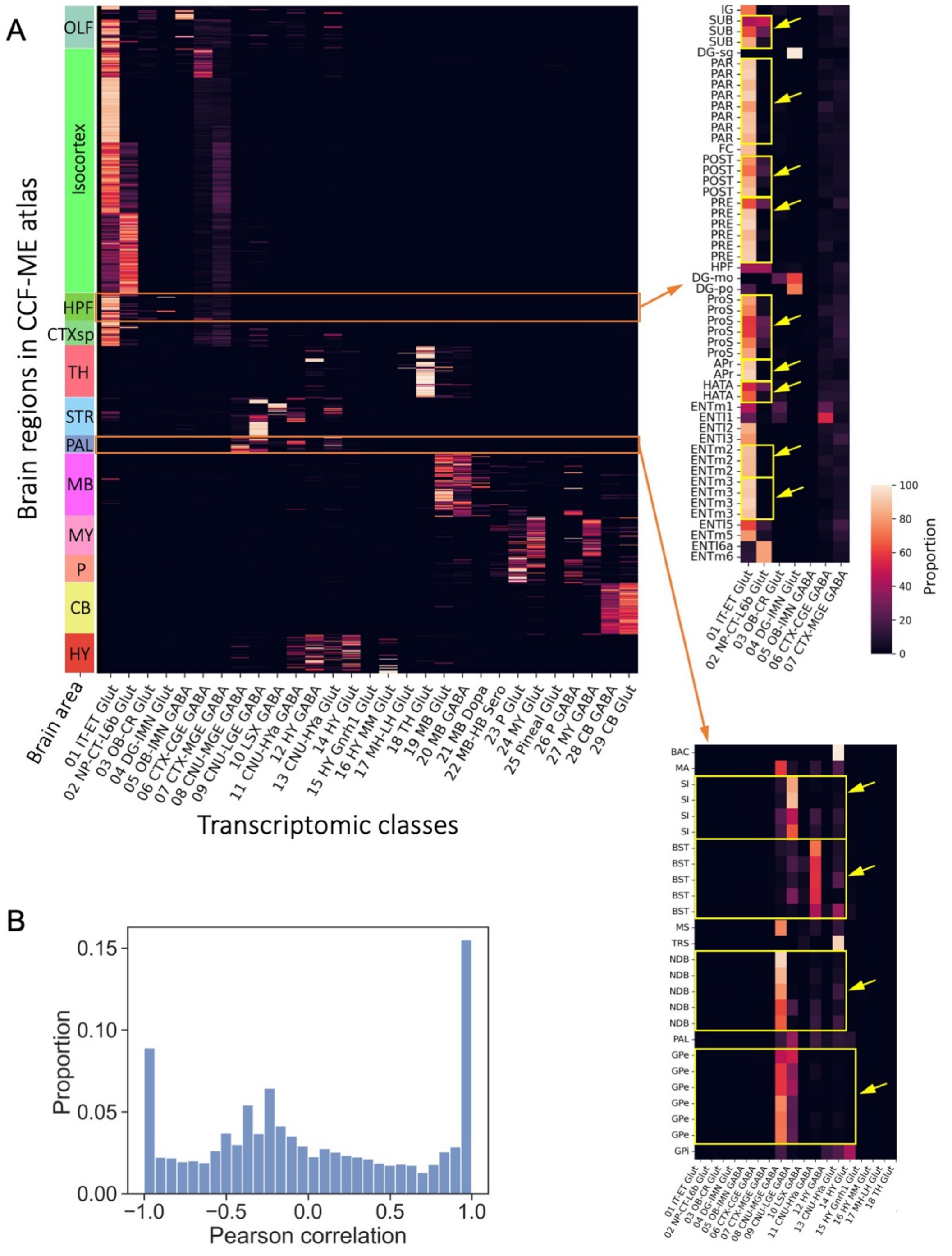

Supplementary Figure S6. Concordance between CCF-ME and transcriptomic data. **A**. Heatmap illustrating the concordance between brain regions in the CCF-ME atlas (left axis) and transcriptomic classes (bottom axis). The color scale indicates the proportion of each transcriptomic class within a given subregion. Major brain areas—including the olfactory regions (OLF), isocortex, hippocampal formation (HPF), cortex-spanning (CTXsp), thalamus (TH), striatum (STR), pallidum (PAL), midbrain (MB), myelencephalon (MY), pons (P), and cerebellum (CB)—are highlighted along the y-axis. The enlarged panels (right) show detailed views of HPF and PAL regions, with highlight of parcellation subregions (indicated by yellow boxes and arrows). **B**. Histogram of Pearson correlation coefficients between CCF-ME subregions within each region based on transcriptomic classes, showing diversified concordance among subregions.

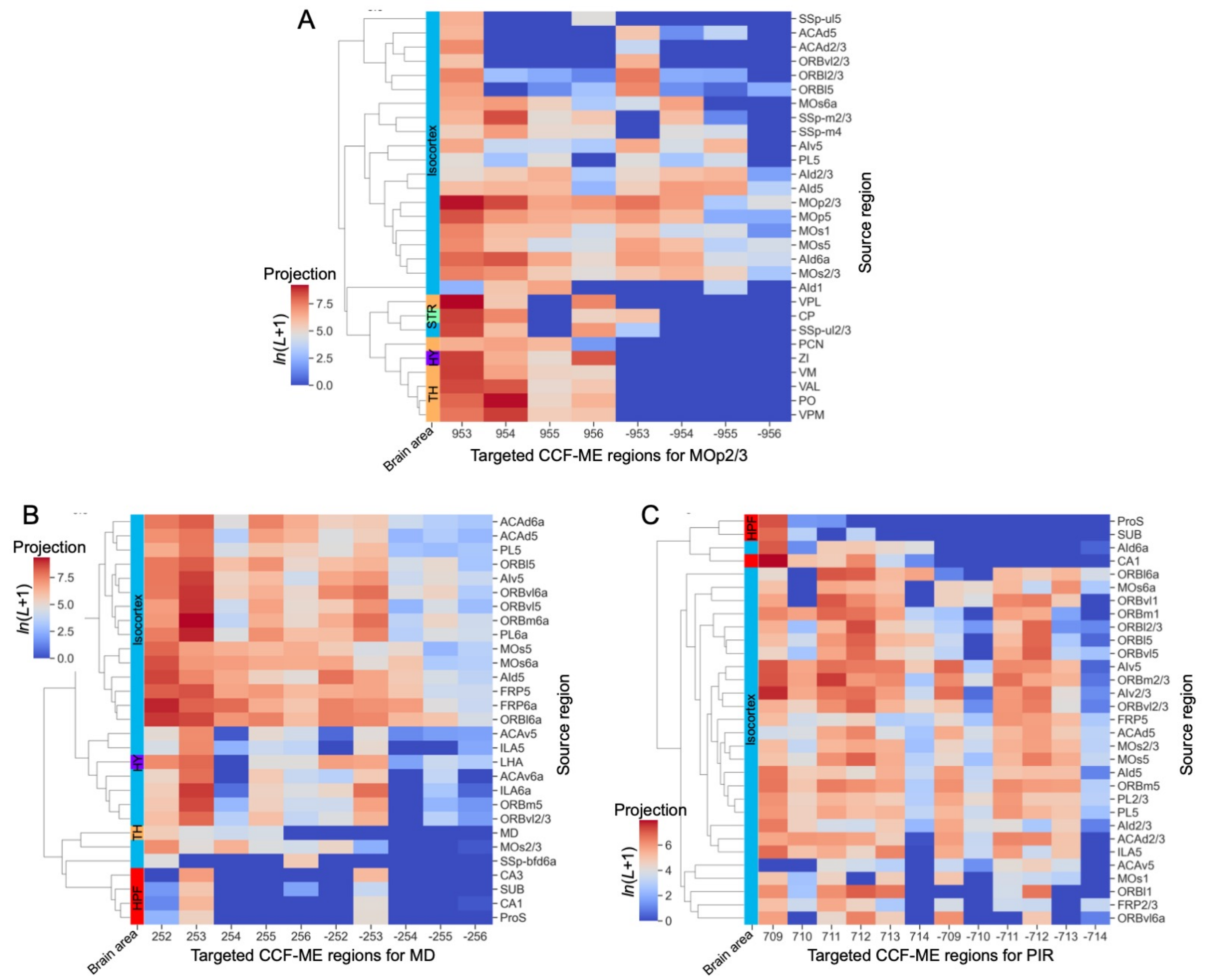

Supplementary Figure S7. Projection specificity among different CCF-ME subregions corresponding to the same CCF region. Each panel shows a clustermap illustrating projections from source regions to CCF-ME subregions of MOp2/3 (A), MD (B), and PIR (C). The left-most column of each panel indicates the brain area for each source region, including the isocortex, hippocampal formation (HPF), thalamus (TH), hypothalamus (HY), and striatum (STR). A negative region ID indicates the projection to contralateral region.

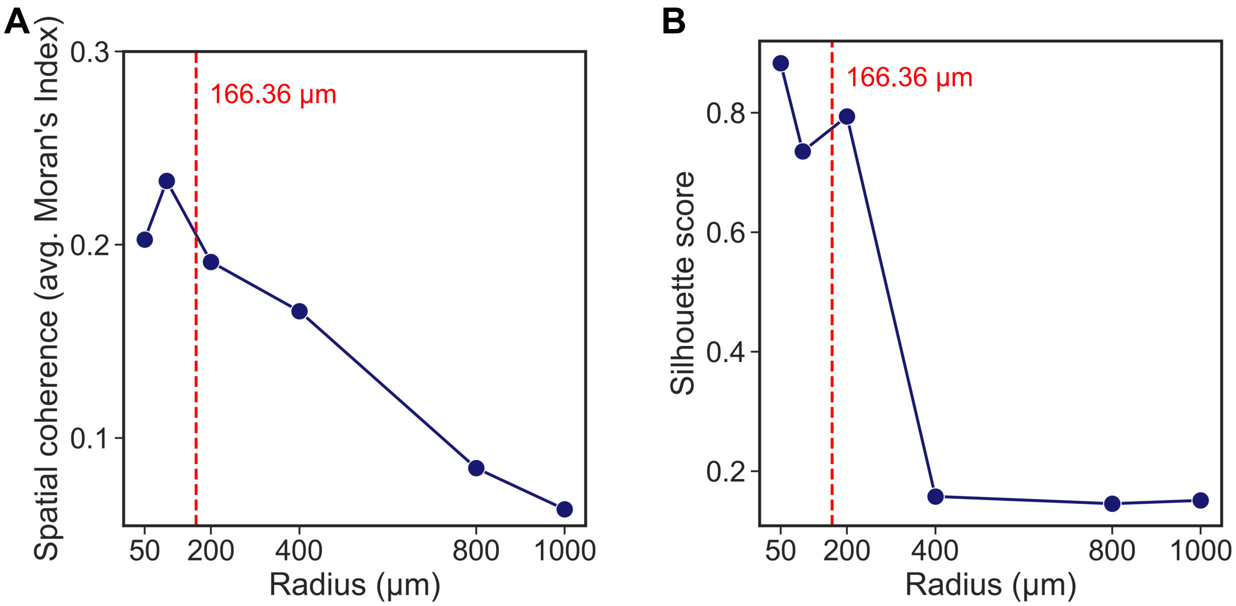

Supplementary Figure S8. Impact of spatial radius and neighbor count on microenvironments. (**A**) Average Moran's Index (spatial coherence) and (**B**) Silhouette score (clustering performance) as functions of spatial radius (50–1000 μm) for shuffled microenvironments (MEs). The radius used in this work (166.36 μm) are highlighted with red dashed lines.
