# Supplementary material for: A mouse brain atlas based on dendritic microenvironments
Source: Nat Neurosci. 2025 Nov 24;29(1):111–22. doi: 10.1038/s41593-025-02119-6 (PMC12779555; doi:10.1038/s41593-025-02119-6)
Supplement: Supplementary file 2 — Reporting Summary [file 41593_2025_2119_MOESM2_ESM.pdf]

nature portfolio

Corresponding author(s): Hanchuan Peng

Last updated by author(s): Sep 7, 2025

Reporting Summary

Nature Portfolio wishes to improve the reproducibility of the work that we publish. This form provides structure for consistency and transparency in reporting. For further information on Nature Portfolio policies, see our Editorial Policies and the Editorial Policy Checklist.

Statistics

For all statistical analyses, confirm that the following items are present in the figure legend, table legend, main text, or Methods section.

n/a	Confirmed	
☐	☒	The exact sample size (*n*) for each experimental group/condition, given as a discrete number and unit of measurement
☐	☒	A statement on whether measurements were taken from distinct samples or whether the same sample was measured repeatedly
☐	☒	The statistical test(s) used AND whether they are one- or two-sided<br>*Only common tests should be described solely by name; describe more complex techniques in the Methods section.*
☒	☐	A description of all covariates tested
☐	☒	A description of any assumptions or corrections, such as tests of normality and adjustment for multiple comparisons
☐	☒	A full description of the statistical parameters including central tendency (e.g. means) or other basic estimates (e.g. regression coefficient) AND variation (e.g. standard deviation) or associated estimates of uncertainty (e.g. confidence intervals)
☐	☒	For null hypothesis testing, the test statistic (e.g. F, t, r) with confidence intervals, effect sizes, degrees of freedom and P value noted<br>*Give P values as exact values whenever suitable.*
☒	☐	For Bayesian analysis, information on the choice of priors and Markov chain Monte Carlo settings
☒	☐	For hierarchical and complex designs, identification of the appropriate level for tests and full reporting of outcomes
☐	☒	Estimates of effect sizes (e.g. Cohen's *d*, Pearson's *r*), indicating how they were calculated

Our web collection on statistics for biologists contains articles on many of the points above.

Software and code

Policy information about availability of computer code

Data collection	The source codes for dendritic morphology reconstruction are available at https://github.com/SEU-ALLEN-codebase/BrainParcellation.
Data analysis	The source codes for morphology evaluation, microenvironment construction, and analyses are available at https://github.com/SEU-ALLEN-codebase/BrainParcellation. The project was built upon our analytical library for neuron morphology pylib (https://github.com/SEU-ALLEN-codebase/pylib). Other dependencies are summarized in the requirement.txt, which can be easily installed through the Python package installer (PIP). Morphological feature extraction was performed using the “global_neuron_feature” plugin of Vaa3D (versions Vaa3D-x 1.1.4 and Vaa3D 4.001). Skeleton resampling was done utilizing the “resample_swc” plugin of Vaa3D (version Vaa3D-x 1.1.4). Short segment pruning was conducted using the “pruning_swc_simple” plugin of Vaa3D (version Vaa3D-x 1.1.4). Feature selection was conducted using pymrmr (version 0.1.1). The clustering of microenvironments for each region was done using the Leiden algorithm (leidenalg; version 0.10.2). Connected component estimation was carried out with cc3d (version 3.12.4).

For manuscripts utilizing custom algorithms or software that are central to the research but not yet described in published literature, software must be made available to editors and reviewers. We strongly encourage code deposition in a community repository (e.g. GitHub). See the Nature Portfolio guidelines for submitting code & software for further information.

Data

Policy information about availability of data

All manuscripts must include a data availability statement. This statement should provide the following information, where applicable:

- Accession codes, unique identifiers, or web links for publicly available datasets
- A description of any restrictions on data availability
- For clinical datasets or third party data, please ensure that the statement adheres to our policy

The reconstructed morphologies and CCF-ME atlas are accessible on Zenodo (https://zenodo.org/records/13761460).

Research involving human participants, their data, or biological material

Policy information about studies with human participants or human data. See also policy information about sex, gender (identity/presentation), and sexual orientation and race, ethnicity and racism.

Reporting on sex and gender	No human participants or related data and materials are used.
Reporting on race, ethnicity, or other socially relevant groupings	No human participants or related data and materials are used.
Population characteristics	No human participants or related data and materials are used.
Recruitment	No human participants or related data and materials are used.
Ethics oversight	No human participants or related data and materials are used.

Note that full information on the approval of the study protocol must also be provided in the manuscript.

Field-specific reporting

Please select the one below that is the best fit for your research. If you are not sure, read the appropriate sections before making your selection.

☒ Life sciences ☐ Behavioural & social sciences ☐ Ecological, evolutionary & environmental sciences

For a reference copy of the document with all sections, see nature.com/documents/nr-reporting-summary-flat.pdf

Life sciences study design

All studies must disclose on these points even when the disclosure is negative.

Sample size	The number of reconstructed neurons (n=101,136) was determined by the throughput of manual annotation of cell bodies, as well as the quality control of the automatic reconstructions.
Data exclusions	All reconstructions in salient brain regions that passed quality control were utilized.
Replication	Attempts at replicating the results were successful using the data and codes we provided.
Randomization	Random sampling of a subset of 5,000 neurons (seed = 1024) was used in evaluating the Moran's Index to speed up calculation for Fig. 2G. Randomization was not applicable for other analyses.
Blinding	Blinding is not applicable to the study design. There was no allocation of treatment and control groups.

Reporting for specific materials, systems and methods

We require information from authors about some types of materials, experimental systems and methods used in many studies. Here, indicate whether each material, system or method listed is relevant to your study. If you are not sure if a list item applies to your research, read the appropriate section before selecting a response.

Materials & experimental systems

n/a	Involved in the study
☒	☐ Antibodies
☒	☐ Eukaryotic cell lines
☒	☐ Palaeontology and archaeology
☒	☐ Animals and other organisms
☒	☐ Clinical data
☒	☐ Dual use research of concern
☒	☐ Plants

Methods

n/a	Involved in the study
☒	☐ ChIP-seq
☒	☐ Flow cytometry
☒	☐ MRI-based neuroimaging

Plants

Seed stocks	No plant was used.
Novel plant genotypes	No plant was used.
Authentication	No plant was used.
